# Genetic determinants of thyroid function in children

**DOI:** 10.1093/ejendo/lvad086

**Published:** 2023-08-02

**Authors:** Tessa A Mulder, Purdey J Campbell, Peter N Taylor, Robin P Peeters, Scott G Wilson, Marco Medici, Colin Dayan, Vincent V W Jaddoe, John P Walsh, Nicholas G Martin, Henning Tiemeier, Tim I M Korevaar

**Affiliations:** Generation R Study Group, Erasmus University Medical Center, Rotterdam, CA 3000, The Netherlands; Department of Internal Medicine, Academic Center for Thyroid Diseases, Erasmus University Medical Center, Rotterdam, CA 3000, The Netherlands; Department of Child and Adolescent Psychiatry, Erasmus University Medical Center, Rotterdam, CA 3000, The Netherlands; Department of Endocrinology & Diabetes, Sir Charles Gairdner Hospital, Nedlands, WA 6009, Australia; Thyroid Research Group, Cardiff University School of Medicine, Cardiff, CF14 4YS, United Kingdom; Department of Internal Medicine, Academic Center for Thyroid Diseases, Erasmus University Medical Center, Rotterdam, CA 3000, The Netherlands; Department of Endocrinology & Diabetes, Sir Charles Gairdner Hospital, Nedlands, WA 6009, Australia; School of Biomedical Sciences, University of Western Australia, Perth, WA 6009, Australia; Department of Twin Research & Genetic Epidemiology, King's College London, London, WC2R 2LS, United Kingdom; Department of Internal Medicine, Academic Center for Thyroid Diseases, Erasmus University Medical Center, Rotterdam, CA 3000, The Netherlands; Center for Endocrine and Diabetes Science, Cardiff University School of Medicine, Cardiff, CF14 4YS, United Kingdom; Generation R Study Group, Erasmus University Medical Center, Rotterdam, CA 3000, The Netherlands; Department of Epidemiology, Erasmus University Medical Center, Rotterdam, 3000 CA, The Netherlands; Department of Endocrinology & Diabetes, Sir Charles Gairdner Hospital, Nedlands, WA 6009, Australia; Medical School, The University of Western Australia, Crawley, WA 6009, Australia; QIMR Berghofer Medical Research Institute, Brisbane, QLD 4006, Australia; Department of Child and Adolescent Psychiatry, Erasmus University Medical Center, Rotterdam, CA 3000, The Netherlands; Department of Social and Behavioral Science, Harvard T.H. Chan School of Public Health, Boston, MA 02115, United States; Department of Internal Medicine, Academic Center for Thyroid Diseases, Erasmus University Medical Center, Rotterdam, CA 3000, The Netherlands

**Keywords:** genetic variants, genetic risk score, thyroid function, childhood

## Abstract

**Objective:**

Genome-wide association studies in adults have identified 42 loci associated with thyroid stimulating hormone (TSH) and 21 loci associated with free thyroxine (FT4) concentrations. While biologically plausible, age-dependent effects have not been assessed. We aimed to study the association of previously identified genetic determinants of TSH and FT4 with TSH and FT4 concentrations in newborns and (pre)school children.

**Methods:**

We selected participants from three population-based prospective cohorts with data on genetic variants and thyroid function: Generation R (*N* = 2169 children, mean age 6 years; *N* = 2388 neonates, the Netherlands), the Avon Longitudinal Study of Parents and Children (ALSPAC; *N* = 3382, age 7.5 years, United Kingdom), and the Brisbane Longitudinal Twin Study (BLTS; *N* = 1680, age 12.1 years, Australia). The association of single nucleotide polymorphisms (SNPs) with TSH and FT4 concentrations was studied with multivariable linear regression models. Weighted polygenic risk scores (PRSs) were defined to combine SNP effects.

**Results:**

In childhood, 30/60 SNPs were associated with TSH and 11/31 SNPs with FT4 after multiple testing correction. The effect sizes for *AADAT, GLIS3, TM4SF4, and VEGFA were* notably larger than in adults. The TSH PRS explained 5.3%-8.4% of the variability in TSH concentrations; the FT4 PRS explained 1.5%-4.2% of the variability in FT4 concentrations. Five TSH SNPs and no FT4 SNPs were associated with thyroid function in neonates.

**Conclusions:**

The effects of many known thyroid function SNPs are already apparent in childhood and some might be notably larger in children as compared to adults. These findings provide new knowledge about genetic regulation of thyroid function in early life.

**Significance**

It is currently unknown whether genetic determinants of TSH and FT4 concentrations in adults have a similar, or any, effect during earlier stages of life. In this study, we investigated associations of adult thyroid function-related genetic determinants with TSH and FT4 concentrations during childhood in three population-based cohorts (*N* = 7231). Overall, we found that 30/60 TSH genetic determinants and 11/31 FT4 genetic determinants identified in adults were also associated with childhood TSH and FT4. Importantly, our results seem to suggest that the effect sizes of some genetic determinants are notably larger in children as compared to adults. Our findings advance the understanding of child thyroid function and aid in untangling the effects of maternal and child thyroid function on offspring outcomes.

## Introduction

Growth and differentiation of many tissues are regulated by thyroid hormone during childhood.^1^ Untreated congenital hypothyroidism causes stunted growth and mental retardation, whereas acquired thyroid dysfunction across childhood leads to more subtle adverse health outcomes.^2^ Hypothyroidism in childhood has been associated with deceleration in growth and skeletal maturation and delayed puberty, whereas hyperthyroidism in childhood has been associated with growth acceleration, advanced bone age, and delayed puberty.^2,3^ Several non-genetic determinants of childhood thyroid function have been identified, such as child age, sex, ethnicity, and anthropometry.^4^ The genetic heritability of thyroid function parameters has been estimated at 65% for thyroid-stimulating hormone (TSH) and 39%-80% for free thyroxine (FT4).^5,6^ In a large genome wide association study (GWAS) in 2018, multiple novel genetic variants of TSH and FT4 concentrations were identified that explain 9.4% of variability in serum TSH and 4.8% of serum FT4 concentrations in adults.^7^ However, it is currently unknown whether genetic determinants of TSH and FT4 concentrations in adults have a similar, or any, effect during earlier stages of life.

Complex and dynamic changes occur in thyroid function across the lifespan.^8^ Most likely, childhood thyroid function is less influenced by autoimmunity, medication usage, comorbidities or aging-related factors that occur throughout life, as compared to adult thyroid function. Therefore, the genetic component of variation in thyroid hormone concentrations may be greater in children as compared to adults. While it seems apparent that the effects of individual genetic variants might be of greater importance if already apparent during childhood, different effects of thyroid hormone on growth and development may warrant age-specific effects of genetic thyroid system determinants. However, there remains a relevant knowledge gap as studies on genetic determinants of childhood thyroid function to date were small, did not report the effects of single genetic variants and have not yet been able to replicate recently discovered loci.^7,9–11^

Therefore, we investigated the association of adult thyroid function-related genetic variants with TSH and FT4 concentrations during childhood in three different population-based cohorts. We hypothesized that only a small number of genetic loci identified in adults would also be associated with childhood serum TSH and FT4 concentrations in the same direction and with the same magnitude of effect.

## Methods

This study was embedded in three prospective birth cohorts: Generation R (The Netherlands), the Avon Longitudinal Study of Parents and Children (ALSPAC, United Kingdom), and the Brisbane Longitudinal Twin Study (BLTS, Australia).

### Study design and participants

In Generation R, all pregnant women with an expected delivery date between April 2002 and January 2006 and living in Rotterdam were invited to participate.^12^ Single nucleotide polymorphism (SNP) data were available for 5732 children in the first wave of data collection and for 1794 children in the second wave. Of the children of European ancestry (*n* = 4315), 2466 children had TSH or FT4 measurements available at the age of 6 years and 2726 children had TSH or FT4 measurements at birth. The Medical Ethics Committee of the Erasmus Medical Centre, Rotterdam, approved the Generation R study.

In ALSPAC, eligible women were those living in the former Avon area in Southwest England, United Kingdom, with an expected delivery date between April 1991 and December 1992. SNP data were available for 7975 unrelated European children. Of these, 3596 children had TSH or FT4 measurements at the age of 7 years. Ethical approval for the study was obtained from the ALSPAC Ethics and Law Committee and the Local Research Ethics Committees.

BLTS was conducted at the Queensland Institute of Medical Research (QIMR) Berghofer, Australia and began in 1992 with approximately 100 pairs of 12-year-old twins (alongside their non-twin siblings and parents) with additional participants recruited annually thereafter.^13^ In BLTS, SNP data were available for 2832 children. Of these, 1746 children had TSH or FT4 measurements available at the age of 12 years. Ethical approval for the study was obtained from the Human Research Ethics Committee of the QIMR Berghofer Medical Research Institute.

Exclusion criteria were pre-existing thyroid disease, thyroid medication, and TSH concentrations outside the cohort-specific 2.5-97.5th centile range. In addition, one child was randomly excluded from each participating sibling pair in Generation R in cord blood and child analyses. Written informed consent was obtained from all participants and/or the children's parents or guardians in all three cohorts. The study was performed in accordance with the principles of the Declaration of Helsinki.

### Genetic variants and risk scores

We extracted data on 60 TSH and 31 FT4 SNPs that were previously identified in adults^7^ from readily available SNP arrays in each cohort. These SNPs were identified in the most recent GWAS that studied both TSH and FT4 SNPs and were independently associated with TSH or FT4 in adults. The 28 TSH SNPs that were identified hereafter^14^ were accessible in Generation R and BLTS only and analysed in a post-hoc analysis.

In Generation R, SNP data were generated in two separate rounds of genotyping and imputation with possible batch effects and are therefore analysed in two separate subsets in this study. ALSPAC and BLTS were also analysed as two separate cohorts.

Unweighted polygenic risk scores (PRSs) were calculated by summation of allele dosages for each SNP. Weighted PRSs were calculated by multiplying the dosage data of each SNP by the previously reported effect estimate in adults.^7^ The weighted and unweighted PRSs were rescaled to range from 0 to 10.

### Thyroid function measurements

In all three cohorts, all participants were invited as part of the follow-up to provide blood samples, and no exclusion criteria were applied beforehand.

In Generation R, serum samples in children were obtained at the age of 6 years, and cord blood samples were obtained directly after birth. Plain tubes were centrifuged, and serum was stored at −80 °C. TSH and FT4 were measured using an electrochemiluminescence immunoassay on the Cobas e601 immunoanalyzer (Roche Diagnostics). The intra- and interassay coefficients of variation were 1.1%-3.0% for TSH at a range of 0.04-0.4 mU L^−1^ and 1.6%-5.0% for FT4 at a range of 1.6-24.1 pmol L^−1^.

In ALSPAC, serum TSH, FT4, and FT3 were measured at age 7 years by chemiluminescent emission using a photomultiplier on Cobas e601 (Roche Diagnostics). The intra-assay coefficients of variation for TSH, FT4, and FT3 were <3.1%, <4%, and <4%, respectively. The interassay coefficients of variation were <7.3%, <6%, and <7%, respectively.

In BLTS, serum TSH, FT4, and FT3 were measured at age 12 years using a chemiluminescent immunoassay on an Abbott ARCHITECT analyser (Abbott Diagnostic).^15^

### Statistical analyses

To make concentrations comparable across cohorts and to approach normality of the residuals, TSH, FT4, and FT3 values were analysed after inverse normal transformation, resulting in SD-scores for the thyroid hormone concentrations. We used multivariable linear regression models to study the association of the single TSH and FT4 SNPs as well as the two respective PRSs with child and neonatal TSH and FT4. Analyses were carried out separately for children and neonates. The explained variance was calculated as the difference in explained variance between the basic model (with covariates) and the full model (including covariates and additionally the PRS).

Assumptions of linear regression models, including linearity, homoscedasticity, and normal distributions of the model residuals, were met for all models in the three cohorts. All models were adjusted for age at blood sampling, child sex, and the first four principal components of genetic ancestry in Caucasians to account for population stratification. In BLTS, we used a linear mixed effect model with a nested random effects structure with separate intercepts for each subject within zygosity group within family to account for relatedness. A symmetric correlation structure between observations at the lowest level of nesting was assumed. Results from the three cohorts were pooled and analysed in a fixed-effects meta-analysis using a two-step approach. To take heterogeneity into account, we subsequently ran models with random effects for SNPs with moderate heterogeneity (*I*^2^ > 30).^16^ However, we expected the effects sizes to be similar across cohorts. Furthermore, the statistical power of a random-effects model is always lower than a fixed-effects model and further decreases with higher heterogeneity.^17^ Therefore, we first used models with fixed effects only and subsequently added random effects in a sensitivity analysis. The genetic data were generated in two runs in Generation R and were therefore analysed as two different subsets (wave 1 and wave 2).

Several additional analyses were performed. First, the analyses of FT4 concentrations were repeated in children with FT4 concentrations in the cohort-specific 2.5-97th centile range. Second, we studied the association of the FT4 SNPs and PRS with FT3 concentrations in children from ALSPAC and BLTS. Third, the TSH and FT4 PRSs were studied as determinants of FT4 and TSH concentrations, respectively. Fourth, we constructed different PRSs including using SNPs associated with thyroid function in childhood, applying estimates from one cohort to another cohort, and excluding SNPs which showed heterogeneity or large deviation from adult estimates. Fifth, we performed a univariate meta-regression with mean age per cohort as predictor and the effect size or the explained variance of FT4 as the dependent variable, using the sample sizes of the cohorts as weights.

To adjust for multiple testing, a false discovery rate (FDR) correction for the number of individual SNPs per outcome was applied (ie, 60 for TSH and 31 for FT4) using the Benjamini and Hochberg method with a q-value threshold of 0.05.^18^ All statistical analyses were performed with R statistical software version 4.2.1 and the R metafor package for the meta-analysis.

Further details on exclusion criteria and measurements are in the Supplemental Methods.

## Results

**Figure 1.**
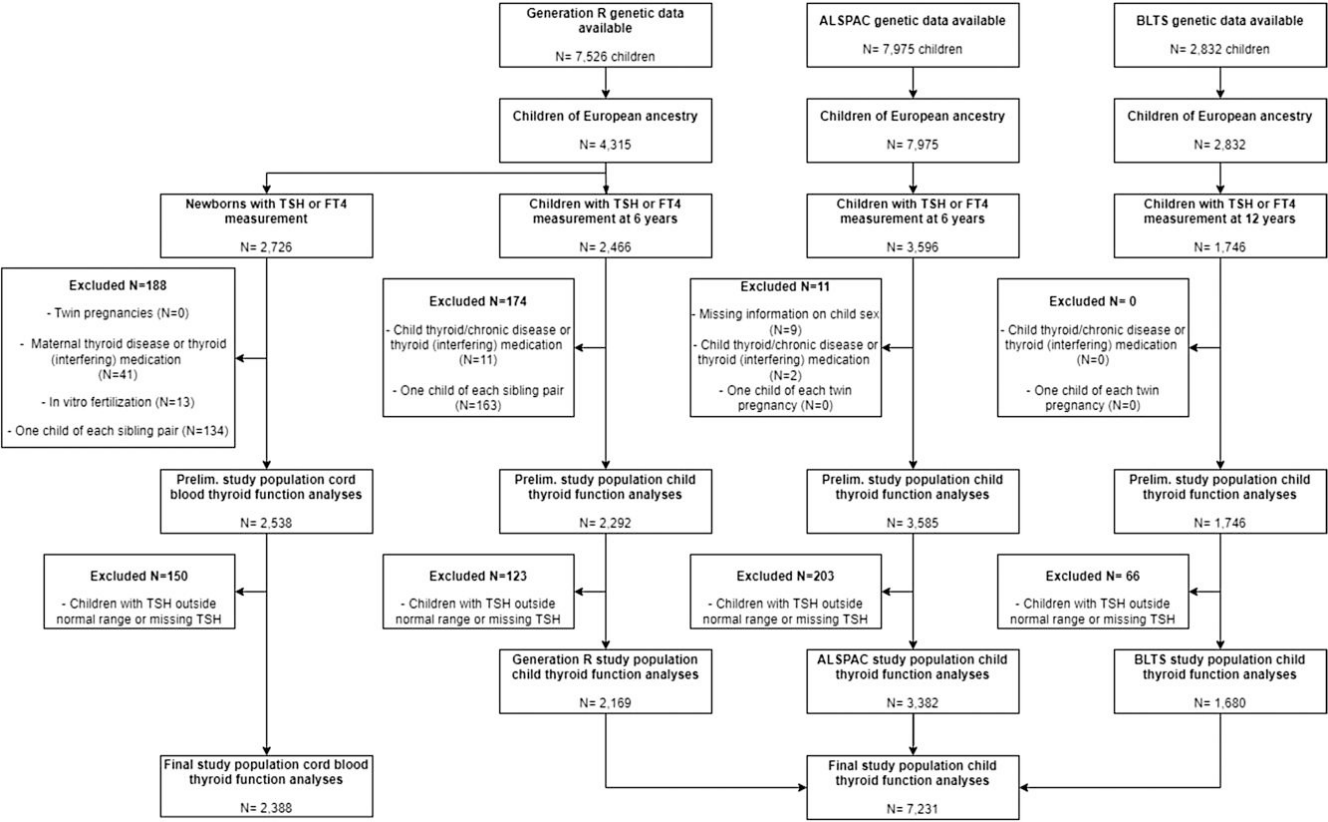
Flowchart of the study population.

The final study population comprised 7231 children in the childhood thyroid function analyses (Figure 1). Descriptive statistics are shown in Table S1. Median (95% range) age at serum thyroid measurements was 6.0 (5.7-7.5) years in Generation R, 7.5 (7.3-8.9) years in ALSPAC, and 12.1 (12.0-12.5) years in BLTS. Median TSH concentrations were 2.4 mU L^−1^ in Generation R, 2.1 mU L^−1^ in ALSPAC, and 1.5 mU L^−1^ in BLTS. Median FT4 concentrations were 16.5 pmol L^−1^ in Generation R, 15.6 pmol L^−1^ in ALSPAC, and 12.6 pmol L^−1^ in BLTS. The minor allele frequencies were similar across cohorts (Table S2). The final study population for newborn thyroid function analyses consisted of 2388 neonates.

### Individual SNPs

**Figure 2.**
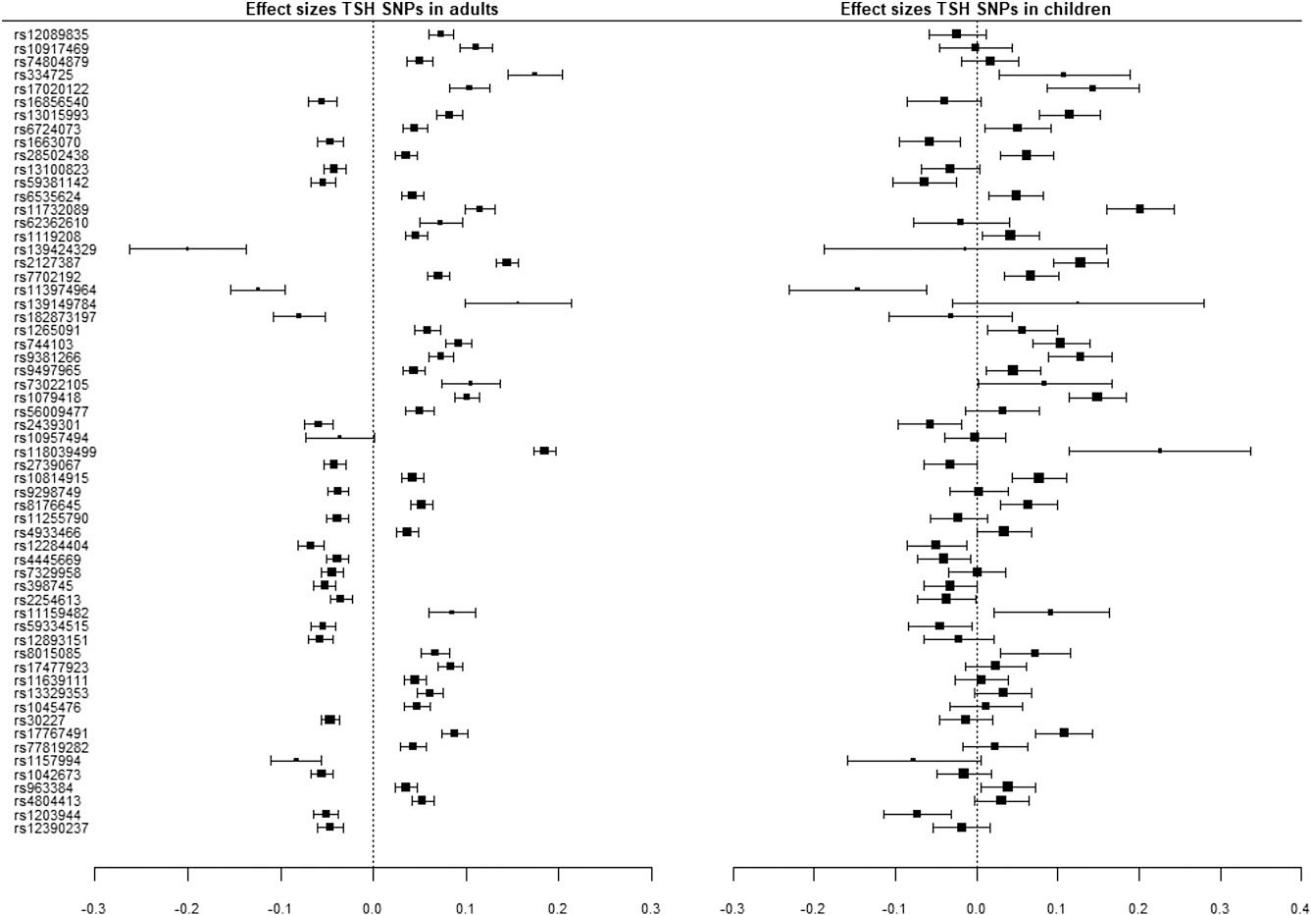
Meta-analysis of FT4 SNPs with TSH concentrations.

**Figure 3.**
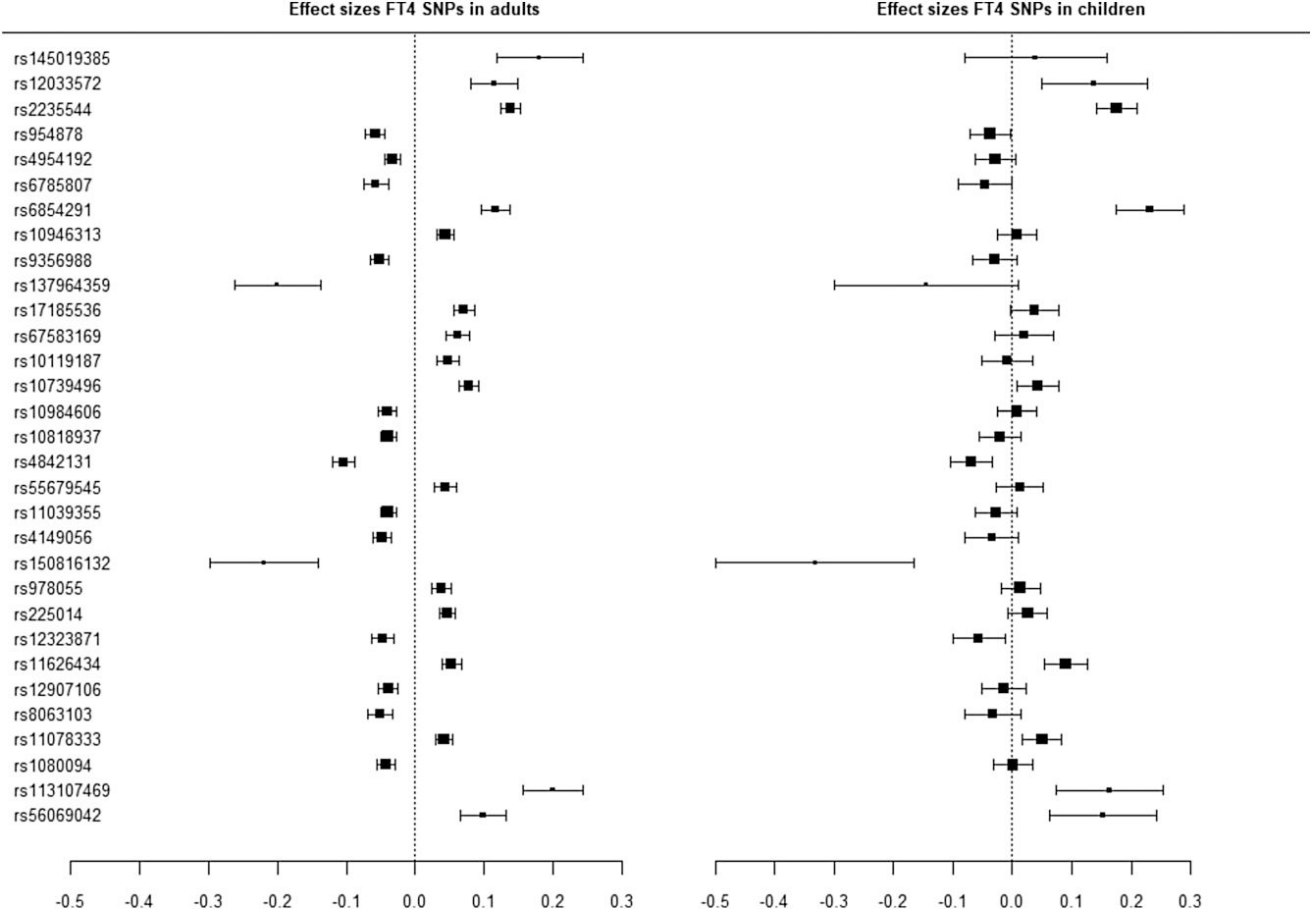
Meta-analysis of FT4 SNPs with FT4 concentrations.

Thirty out of 60 SNPs were associated with childhood TSH concentrations and 11 out of 31 SNPs with childhood FT4 concentrations after correction for multiple testing (Tables 1 and 2, Figures 2 and 3). The direction of effect was similar to adults for 55 out of 60 TSH SNPs and 28 out of 31 FT4 SNPs. None of the SNPs showed an inverse direction of effect in all cohorts as compared to adults (Tables S3 and S4).

**Table 1.** Meta-analysis of TSH SNPs with TSH concentrations.

SNP	Chr	Position	Locus	A1	A2	b Adults	b	Deviation from adults in %	se	se adults	pval	p.fdr	*I* ^2^	CQp
rs12089835	1	19771438	CAPZB	t	c	0.073	−0.024	−132.9	0.018	0.007	.17	.23	0	0.84
rs10917469	1	19843576	CAPZB	a	g	0.111	−0.001	−100.9	0.023	0.009	.97	.97	0	0.64
rs74804879	1	19862320	CAPZB	t	c	0.050	0.017	−66.0	0.018	0.007	.35	.43	0	0.83
rs334725	1	61610049	NFIA	a	g	0.174	0.108	−37.9	0.041	0.015	.01	.02^a^	42	0.16
rs17020122	1	108357391	VAV3	t	c	0.104	0.143	37.5	0.029	0.011	<.01	<.001	5	0.37
rs16856540	2	217580413	IGFBP5	t	c	−0.055	−0.04	−27.3	0.023	0.008	.09	.14	17	0.31
rs13015993	2	217625523	IGFBP5	a	g	0.082	0.115	40.2	0.019	0.007	<.001	<.001	0	0.77
rs6724073	2	218236786	DIRC3	t	c	0.045	0.051	13.3	0.021	0.007	.02	.04^a^	68	0.03
rs1663070	3	12239852	SYN2	t	c	−0.046	−0.058	26.1	0.019	0.007	<.01	.01	12	0.33
rs28502438	3	149220109	TM4SF4	t	c	0.035	0.062	77.1	0.017	0.006	<.001	<.01	0	0.47
rs13100823	3	185514088	IGF2BP2	t	c	−0.042	−0.032	−23.8	0.018	0.006	.08	.13	42	0.16
rs59381142	3	193916181	HES1	a	g	−0.054	−0.064	18.5	0.020	0.007	<.01	<.01	0	0.79
rs6535624	4	149587905	NR3C2	a	g	0.042	0.049	16.7	0.017	0.006	<.01	.01^a^	34	0.21
rs11732089	4	149665602	NR3C2	t	c	0.115	0.202	75.7	0.021	0.008	<.001	<.001	27	0.25
rs62362610	5	76439961	PDE8B	c	g	0.073	−0.019	−126.0	0.030	0.012	.54	.61	0	0.83
rs1119208	5	76488613	PDE8B	t	c	0.046	0.042	−8.7	0.018	0.006	.02	.04	0	0.64
rs139424329	5	76 495 539	PDE8B	a	g	−0.200	−0.014	−93.0	0.089	0.032	.87	.92	0	0.92
rs2127387	5	76532571	PDE8B	a	g	0.144	0.128	−11.1	0.017	0.006	<.001	<.001	41	0.17
rs7702192	5	76554807	PDE8B	a	c	0.070	0.067	−4.3	0.017	0.006	<.001	<.001	0	0.86
rs113974964	5	76652403	PDE8B	t	c	−0.124	−0.146	17.7	0.043	0.015	<.01	<.01	0	0.68
rs139149784	5	76660193	PDE8B	a	g	0.156	0.125	−19.9	0.079	0.029	.11	.17	46	0.13
rs182873197	5	76773148	PDE8B	t	c	−0.080	−0.032	−60.0	0.039	0.014	.41	.48	0	0.50
rs1265091	6	31108129	PSORS1C1	t	c	0.058	0.056	−3.4	0.022	0.007	.01	.03	39	0.18
rs744103	6	43805362	VEGFA/LOC100132354	a	t	0.092	0.104	13.0	0.018	0.007	<.001	<.001	0	0.82
rs9381266	6	43905037	VEGFA/LOC100132354	t	c	0.073	0.128	75.3	0.020	0.007	<.001	<.001	0	0.45
rs9497965	6	148521292	SASH1	t	c	0.044	0.045	2.3	0.017	0.006	.01	.03^a^	51	0.10
rs73022105	6	165973757	PDE10A	t	c	0.105	0.084	−20.0	0.042	0.016	<.05	.09	0	0.50
rs1079418	6	166047034	PDE10A	a	g	0.101	0.149	47.5	0.018	0.007	<.001	<.001	74	0.01
rs56009477	8	23356964	SLC25A37	a	g	0.050	0.032	−36.0	0.023	0.008	.17	.23	0	0.94
rs2439301	8	32433013	NRG1	a	g	−0.059	−0.057	−3.4	0.020	0.008	<.01	.01	0	0.78
rs10957494	8	70365025	SULF1	a	g	−0.036	−0.002	−94.4	0.019	0.020	.91	.95	0	0.98
rs118039499	8	133771635	TG	a	c	0.185	0.226	22.2	0.057	0.006	<.001	<.001	0	0.83
rs2739067	8	133951991	TG	a	g	−0.042	−0.032	−23.8	0.017	0.006	.06	.11	0	0.58
rs10814915	9	4290544	GLIS3	t	c	0.042	0.077	83.3	0.017	0.006	<.001	<.001	0	0.45
rs9298749	9	16214340	C9orf92	a	c	−0.038	0.003	−107.9	0.018	0.006	.86	.92	14	0.32
rs8176645	9	136149098	ABO	a	t	0.052	0.064	23.1	0.018	0.006	<.001	<.02	0	0.40
rs11255790	10	8682180	GATA3	t	c	−0.039	−0.022	−43.6	0.018	0.006	.24	.32	23	0.28
rs4933466	10	89849519	PTEN	a	g	0.037	0.034	−8.1	0.017	0.006	<.05	.09	0	0.39
rs12284404	11	45228686	PRDM11	a	g	−0.067	−0.049	−26.9	0.019	0.007	.01	.02	0	0.77
rs4445669	11	115045237	CADM1	t	c	−0.039	−0.04	2.6	0.017	0.006	.02	.04^a^	53	0.09
rs7329958	13	24782080	SPATA13	t	c	−0.044	0.001	−102.3	0.018	0.006	.94	.95	7	0.36
rs398745	14	36536181	MBIP	a	c	−0.052	−0.032	−38.5	0.017	0.006	.06	.11	48	0.12
rs2254613	14	36713154	MBIP	t	g	−0.035	−0.037	5.7	0.018	0.006	.04	.08	0	0.44
rs11159482	14	81490842	TSHR	t	c	0.085	0.092	8.2	0.036	0.013	.01	.03^a^	53	0.09
rs59334515	14	81594143	TSHR	t	c	−0.054	−0.045	−16.7	0.020	0.007	.02	<.05	14	0.32
rs12893151	14	81619945	TSHR	a	c	−0.057	−0.022	−61.4	0.022	0.007	.31	.39	0	0.40
rs8015085	14	93585331	ITPK1	a	g	0.067	0.072	7.5	0.022	0.008	<.01	<.01	44	0.15
rs17477923	15	49711185	FAM227B/FGF7	t	c	0.083	0.024	−71.1	0.019	0.007	.22	.30	48	0.12
rs11639111	15	49749735	FAM227B/FGF7	t	c	0.045	0.006	−86.7	0.017	0.006	.71	.78	0	0.98
rs13329353	15	89113877	DET1	t	c	0.061	0.033	−45.9	0.018	0.007	.07	.11	0	0.40
rs1045476	16	4015313	ADCY9	a	g	0.047	0.012	−74.5	0.023	0.007	.60	.66	67	0.03
rs30227	16	14405428	MIR365A	t	c	−0.046	−0.013	−71.7	0.017	0.005	.44	.51	0	0.85
rs17767491	16	79745487	MAF	a	g	0.088	0.108	22.7	0.018	0.007	<.001	<.001	0	0.48
rs77819282	17	44762589	NSF	a	g	0.043	0.023	−46.5	0.020	0.007	.25	.32	24	0.27
rs1157994	17	59338574	BCAS3	a	g	−0.083	−0.077	−7.2	0.042	0.014	.07	.11	0	0.61
rs1042673	17	70121339	SOX9	a	g	−0.055	−0.015	−72.7	0.017	0.006	.39	.47	0	0.55
rs963384	17	70369758	SOX9	t	c	0.035	0.039	11.4	0.017	0.006	.02	<.05	0	0.89
rs4804413	19	7222655	INSR	t	c	0.053	0.031	−41.5	0.017	0.006	.06	.11	42	0.16
rs1203944	20	22596879	FOXA2	t	c	−0.051	−0.073	43.1	0.021	0.007	<.001	<.01	0	0.44
rs12390237	23	3612081	PRKX	a	g	−0.046	−0.018	−60.9	0.018	0.007	.31	.39	58	0.06

Abbreviations: A1, Allele 1; A2, Allele 2; b, beta A1; Chr, chromosome; CQp, *P*-value of Cochran's *Q* test for heterogeneity; *I*^2^, percentage of variance that is attributable to study heterogeneity; p.fdr, false discovery rate corrected *P*-value; pval, *P*-value; se, standard error; SNP, single nucleotide polymorphism.

a p.fdr >.05 in model with random intercepts.

**Table 2.** Meta-analysis FT4 SNPs with FT4 concentrations.

SNP	Chr	Position	Locus	A1	A2	b adults	b	Deviation from adults in %	se	se adults	pval	p.fdr	*I* ^2^	CQp
rs145019385	1	54252139	DIO1	t	c	0.181	0.039	−78.5	0.061	0.032	.52	.61	16	0.31
rs12033572	1	54369674	DIO1	c	g	0.115	0.138	20.0	0.045	0.017	<.01	.01	12	0.33
rs2235544	1	54375570	DIO1	a	c	0.139	0.176	26.6	0.017	0.007	<.001	<.001	71	0.02
rs954878	1	54578401	DIO1	a	g	−0.058	−0.037	−36.2	0.017	0.007	.03	.07	72	0.01
rs4954192	2	1.36E+08	ACMSD	t	c	−0.033	−0.028	−15.2	0.017	0.006	.11	.20	34	0.21
rs6785807	3	1.82E+08	SOX2-OT	a	g	−0.057	−0.046	−19.3	0.023	0.009	<.05	.11	0	0.60
rs6854291	4	1.71E+08	AADAT	a	g	0.117	0.232	98.3	0.029	0.011	<.001	<.001	63	<0.05
rs10946313	6	19381386	ID4	t	c	0.044	0.008	−81.8	0.017	0.006	.66	.71	0	0.60
rs9356988	6	25777481	SLC17A4	a	g	−0.052	−0.029	−44.2	0.019	0.007	.13	.22	0	0.70
rs137964359	6	26001742	SLC17A4	t	c	−0.200	−0.144	−28.0	0.079	0.032	.07	.16	0	0.45
rs17185536	6	1.01E+08	LOC728012	t	c	0.071	0.037	−47.9	0.021	0.008	.08	.16	25	0.26
rs67583169	8	61212179	CA8	c	g	0.062	0.02	−67.7	0.025	0.009	.41	.53	5	0.37
rs10119187	9	4223660	GLIS3	t	c	0.048	−0.008	−116.7	0.022	0.008	.73	.75	37	0.19
rs10739496	9	1.01E+08	FOXE1	t	c	0.078	0.043	−44.9	0.018	0.007	.02	.04	0	0.89
rs10984606	9	1.01E+08	FOXE1	t	g	−0.040	0.008	−120.0	0.017	0.007	.64	.71	0	0.81
rs10818937	9	1.27E+08	NEK6	t	c	−0.039	−0.02	−48.7	0.018	0.006	.26	.37	4	0.37
rs4842131	9	1.39E+08	LHX3	t	c	−0.104	−0.069	−33.7	0.018	0.008	<.001	<.01	0	0.88
rs55679545	9	1.39E+08	LHX3	a	g	0.044	0.013	−70.5	0.020	0.008	.53	.61	0	0.41
rs11039355	11	47737501	FNBP4	t	c	−0.039	−0.027	−30.8	0.018	0.006	.13	.22	0	0.64
rs4149056	12	21331549	SLCO1B1	t	c	−0.048	−0.034	−29.2	0.023	0.007	.14	.22	67	0.03
rs150816132	14	80464293	DIO2	a	g	−0.220	−0.332	50.9	0.085	0.040	<.001	<.01	0	0.77
rs978055	14	80534869	DIO2	a	t	0.038	0.014	−63.2	0.017	0.007	.41	.53	58	0.07
rs225014	14	80669580	DIO2	t	c	0.047	0.026	−44.7	0.017	0.006	.14	.22	0	0.53
rs12323871	14	1.02E+08	DIO3OS	t	c	−0.047	−0.056	19.1	0.022	0.008	.01	.03	45	0.14
rs11626434	14	1.02E+08	DIO3OS	c	g	0.053	0.09	69.8	0.018	0.007	<.001	<.001	0	0.52
rs12907106	15	63873658	USP3	c	g	−0.039	−0.014	−64.1	0.019	0.007	.45	.56	0	0.96
rs8063103	16	12703395	SNX29	c	g	−0.051	−0.033	−35.3	0.024	0.009	.17	.25	8	0.35
rs11078333	17	16049626	NCOR1	a	t	0.042	0.05	19.0	0.017	0.006	<.01	.01	0	0.58
rs1080094	18	29173795	SLC25A52	a	g	−0.042	0.001	−102.4	0.017	0.007	.94	.94	0	0.50
rs113107469	18	29306737	SLC25A52	t	c	0.200	0.163	−18.5	0.046	0.022	<.001	<.01^a^	80	<0.01
rs56069042	18	57914644	MC4R	a	g	0.099	0.152	53.5	0.046	0.017	<.01	<.01	18	0.30

Abbreviations: A1, Allele 1; A2, Allele 2; b, beta A1; Chr, chromosome; CQp, *P*-value of Cochran's *Q* test for heterogeneity; *I*^2^, percentage of variance that is attributable to study heterogeneity; p.fdr, false discovery rate corrected *P*-value; pval, *P*-value; se, standard error; SNP, single nucleotide polymorphism.

a p.fdr >.05 in model with random intercepts.

For 29 out of 60 TSH SNPs and 9 out of 31 FT4 SNPs, the effects estimates were within ± 30% deviation (arbitrary cutoff to provide an indication) from the adult effect estimates (Tables 1 and 2). Twenty-three (77%) SNPs associated with childhood TSH and 8 (73%) associated with childhood FT4 exceeded the adult effect estimate. The FT4-associated SNP in the *AADAT* locus and three TSH-associated SNPs in the *GLIS3*, *TM4SF4*, and *VEGFA* locus had effect estimates that were notably larger than those in adults.

When SNPs with an *I*^2^ value >30 were analysed using a random effects model, 6 out of 30 TSH SNPs, and 1 out of 11 FT4 SNPs that were statistically significant in the fixed effects model were not associated with TSH or FT4 anymore (Tables 1 and 2).

In newborns, 5 TSH SNPs were associated with TSH concentrations, and these SNPs were also associated with TSH in childhood (Table S5 and Table 1). None of the FT4 SNPs identified in adults were associated with FT4 concentrations in newborns (Table S6).

### PRSs

The weighted PRS including TSH SNPs was associated with TSH concentrations (β = .16, SE = 0.01, *P* ≤ .001) (Table 3). The variation in TSH explained by this PRS was 5.7% and 8.4% in Generation R, 5.3% in ALSPAC, and 6.7% in BLTS. The TSH-PRS was also associated with FT4 concentrations (β = −.04, SE = 0.01, *P≤* .001) and per study explained 0.1%-0.6% of the variation in FT4 concentrations (Table 3).

**Table 3.** Associations of TSH polygenic risk score (PRS) with thyroid function in childhood.

		b	se	pval	*I* ^2^ (meta-analysis^a^) or Explained Variance (cohort)	*N*
Meta-analysis	TSH	0.16	0.01	<.001	7	7231
	FT4	−0.04	0.01	<.001	0	7206
Generation R wave 1	TSH	0.18	0.02	<.001	0.057	1542
	FT4	−0.05	0.02	<.001	0.005	1534
Generation R wave 2	TSH	0.18	0.02	<.001	0.084	627
	FT4	−0.04	0.02	.12	0.004	625
ALSPAC	TSH	0.15	0.01	<.001	0.053	3382
	FT4	−0.03	0.01	.01	0.002	3367
BLTS	TSH	0.17	0.02	<.001	0.067	1680
	FT4	−0.05	0.02	<.01	0.006	1680

Abbreviation: *I*^2^, percentage of variance that is attributable to study heterogeneity.

a *P*-values of Cochran's *Q* test for heterogeneity .36 for TSH and .72 for FT4.

The weighted PRS including FT4 SNPs was associated with FT4 concentrations (β = .12, SE = 0.01, *P* ≤ .001) (Table 4). The variation in FT4 explained by this PRS was 4.2% and 3.8% in Generation R, 2.9% in ALSPAC, and 1.5% in BLTS. The FT4-PRS was also associated with TSH concentrations (β = .03, SE = 0.01, *P* ≤ .001) and per study explained 0.0%-0.3% of the variation in TSH concentrations (Table 4).

**Table 4.** Associations of FT4 polygenic risk score (PRS) with thyroid function in childhood.

		b	se	pval	*I* ^2^ (meta-analysis^a^) or explained variance (cohort)	*N*
Meta-analysis	FT4	0.12	0.01	<.001	24	7206
	FT4 normal range	0.12	0.01	<.001	24	6894
	TSH	0.03	0.01	<.001	0	7231
	FT3	−0.04	0.01	<.001	0	5023
Generation R wave 1	FT4	0.14	0.02	<.001	0.042	1534
	FT4 normal range	0.14	0.02	<.001	0.047	1470
	TSH	0.03	0.02	.14	0.001	1542
Generation R wave 2	FT4	0.12	0.02	<.001	0.038	625
	FT4 normal range	0.10	0.02	<.001	0.028	590
	TSH	0.01	0.02	.58	0.00	627
ALSPAC	FT4	0.13	0.01	<.001	0.029	3367
	FT4 normal range	0.12	0.01	<.001	0.022	3222
	TSH	0.04	0.01	<.01	0.003	3382
	FT3	−0.04	0.01	<.01	0.002	3343
BLTS	FT4	0.09	0.02	<.001	0.015	1680
	FT4 normal range	0.09	0.02	<.001	0.015	1612
	TSH	0.02	0.02	.21	0.001	1680
	FT3	−0.04	0.02	.03	0.003	1680

Abbreviation: *I*^2^, percentage of variance that is attributable to study heterogeneity.

a *P*-values of Cochran's *Q* test for heterogeneity .27 for FT4 and FT4 normal range,.53 for TSH, and >.99 for FT3.

The weighted PRS including TSH SNPs was associated with TSH concentrations in newborns, whereas the FT4 PRS was not associated with FT4 concentrations in newborns (Table S7).

The effect estimates for unweighted PRSs were comparable to the weighted PRSs, albeit with lower explained variabilities for the variation in TSH and FT4 (Tables S8 and S9).

### Additional analyses

Four FT4 SNPs were associated with FT3 concentrations after multiple testing correction, but three of those showed substantial heterogeneity with I-squared values >80 and none were associated with FT3 in models with random intercepts (Table 5). However, 8 SNPs were associated with FT3 in ALSPAC only (Table S10). The FT4 PRS was associated with FT3 concentrations (β = −.04, SE = 0.01, *P*≤.001) and the explained FT3 variability was 0.2% in ALSPAC and 0.3% in BLTS (Table 4).

**Table 5.** Meta-analysis FT4 SNPs with FT3 concentrations.

SNP	Chr	Position	Locus	A1	A2	B	se	pval	p.fdr	*I* ^2^	CQp
rs145019385	1	54252139	DIO1	t	c	0.036	0.070	.61	.85	37	0.21
rs12033572	1	54369674	DIO1	c	g	0.019	0.051	.70	.87	74	<0.05
rs2235544	1	54375570	DIO1	a	c	0.094	0.020	<.001	<.001^a^	98	<0.001
rs954878	1	54578401	DIO1	a	g	−0.048	0.020	.02	.08	83	0.01
rs4954192	2	1.36E+08	ACMSD	t	c	−0.047	0.021	.02	.08	0	0.84
rs6785807	3	1.82E+08	SOX2-OT	a	g	−0.026	0.027	.34	.62	0	0.45
rs6854291	4	1.71E+08	AADAT	a	g	0.101	0.036	<.01	.033^a^	95	<0.001
rs10946313	6	19381386	ID4	t	c	0.023	0.021	.28	.54	0	0.73
rs9356988	6	25777481	SLC17A4	a	g	−0.005	0.023	.83	.88	0	0.37
rs137964359	6	26001742	SLC17A4	t	c	−0.051	0.085	.55	.81	0	0.60
rs17185536	6	1.01E+08	LOC728012	t	c	0.073	0.025	<.01	.033^a^	0	0.68
rs67583169	8	61212179	CA8	c	g	0.013	0.030	.66	.85	0	>0.99
rs10119187	9	4223660	GLIS3	t	c	−0.023	0.027	.40	.69	0	0.91
rs10739496	9	1.01E+08	FOXE1	t	c	0.006	0.022	.77	.88	91	<0.01
rs10984606	9	1.01E+08	FOXE1	t	g	0.047	0.020	.02	.08	75	<0.05
rs10818937	9	1.27E+08	NEK6	t	c	0.014	0.022	.51	.80	0	0.52
rs4842131	9	1.39E+08	LHX3	t	c	−0.056	0.021	.01	.06	0	0.34
rs55679545	9	1.39E+08	LHX3	a	g	0.019	0.024	.43	.71	0	0.82
rs11039355	11	47737501	FNBP4	t	c	−0.007	0.022	.74	.88	79	0.03
rs4149056	12	21331549	SLCO1B1	t	c	−0.037	0.029	.20	.41	0	0.84
rs150816132	14	80464293	DIO2	a	g	−0.146	0.103	.16	.35	87	0.01
rs978055	14	80534869	DIO2	a	t	−0.010	0.021	.64	.85	65	0.09
rs225014	14	80669580	DIO2	t	c	−0.003	0.021	.88	.91	0	0.79
rs12323871	14	1.02E+08	DIO3OS	t	c	−0.060	0.026	.02	.08	0	0.73
rs11626434	14	1.02E+08	DIO3OS	c	g	0.038	0.021	.07	.19	82	0.02
rs12907106	15	63873658	USP3	c	g	−0.002	0.023	.92	.92	37	0.21
rs8063103	16	12703395	SNX29	c	g	−0.042	0.029	.15	.35	82	0.02
rs11078333	17	16049626	NCOR1	a	t	0.046	0.021	.03	.08	49	0.16
rs1080094	18	29173795	SLC25A52	a	g	0.005	0.021	.81	.88	0	0.68
rs113107469	18	29306737	SLC25A52	t	c	0.234	0.055	<.001	<.001^a^	88	<0.01
rs56069042	18	57914644	MC4R	a	g	0.108	0.058	.06	.18	0	0.44

Abbreviations: A1, Allele 1; A2, Allele 2; b, beta A1; Chr, chromosome; CQp, *P*-value of Cochran's *Q* test for heterogeneity; *I*^2^, percentage of variance that is attributable to study heterogeneity; p.fdr, false discovery rate corrected *P*-value; pval, *P*-value; se, standard error; SNP, single nucleotide polymorphism.

a p.fdr >.05 in model with random intercepts.

Results were similar after excluding FT4 concentrations outside the normal range (Tables S11 and S12).

Small differences in explained variance were apparent when the PRS was constructed using different approaches (Table S13). The explained variance increased in all cohorts when only including SNPs that were associated with TSH or FT4 in childhood, ranging 6.5%-7.4% instead of 5.3%-6.7% for TSH and 2.0%-5.4% instead of 1.5%-4.2% for FT4. When only SNPs within ± 30% deviation from adults or SNPs without heterogeneity were included, the explained variance decreased. The clearest decline was found in ALSPAC and Generation R for FT4, in which the explained variances changed from 2.9% to 0.8% and from 4.2% to 1.5%, respectively.

Three out of 28 SNPs that were discovered after the previously mentioned GWAS were associated with TSH concentrations in childhood (Tables S14 and S15).

There was a negative association of age with effect size and explained variance of the FT4 PRS with FT4 concentrations (explained variance: β = −.39, SE = 0.09, *P* = .046; effect size: β = −.01, SE = 0.00, *P* = .053; Table S16), although the latter was not statistically significant.

## Discussion

In this meta-analysis of three population-based prospective cohorts, 30 out of 60 TSH SNPs and 11 out of 31 FT4 SNPs identified in adults were also associated with childhood TSH and FT4. The weighted polygenic risk score (PRS) including TSH SNPs explained 5.3%-8.4% of childhood TSH variability, whereas the FT4 PRS explained 1.5%-4.2% of childhood FT4 variability. Furthermore, we identified five SNPs associated with TSH at birth. Our results seem to suggest that the effect sizes of some SNPs are notably larger in children as compared to adults.

In the current study, multiple SNPs that were associated with thyroid function in adults were also associated with TSH or FT4 during childhood. The explained variability in FT4 concentrations as assessed by the PRS differed between the cohorts, with what seemed to follow a trend of a lower explained variability with a higher median age of the cohort and this finding is supported by a negative association of age with the explained variance. In line with this, thyroid function is likely to be less explained by genetics and more variable within a population as participants get older, accumulate exposure to environmental factors and acquire thyroid autoimmunity. In our study, however, the explained variability of TSH and FT4 as assessed by the PRS including the same SNPs as in adults was lower than in adults (ie, 9.4% for TSH and 4.8% for FT4 in adults). A possible explanation is that the SNPs that we studied were identified in adults of which some might be important for auto-immunity, which is likely less involved in childhood thyroid function. Moreover, there might be age-specific effects of genetic thyroid system determinants that are only apparent in childhood and therefore were not identified in adults and subsequently not investigated in our study. Therefore, future studies should include a GWAS on childhood thyroid function to identify childhood-specific genetic determinants of thyroid function. In addition, future studies should investigate gene-age interactions and the role of epigenetic regulation. This could help to increase the explained variability of TSH and FT4.

Importantly, there could be other factors than age contributing to the difference in explained variability of FT4 concentrations between the cohorts, such as iodine status. Iodine intake in the Netherlands (Generation R) is adequate, whereas the United Kingdom (ALSPAC) is moderately iodine-deficient and iodine deficiency re-emerged in Australia in the 1990s when BLTS participants were recruited.^19–21^ The contribution of iodine status to the variation in FT4 concentrations as compared to the genetic contribution might be higher in countries with a median insufficient iodine status and a wider range of iodine levels, as compared to countries with an adequate iodine intake. Indeed, the highest explained variability in FT4 concentrations was observed in Generation R, whereas the explained variability was lower in ALSPAC and BLTS.

Since childhood estimates from previous studies were not available, we were limited by using adult estimates for generating PRSs. Calculating PRSs with different methods did not yield large differences in most analyses. The decline in explained variance of FT4 when only SNPs without heterogeneity were included in the PRS in Generation R and ALSPAC (from 4.2% and 2.9% to 1.5% and 0.8%, respectively) can be explained by the fact that three out of five excluded SNPs had large effect estimates in adults, Generation R and/or ALSPAC and thus contributed to a relative large extent to the explained variance. The effect estimates of these SNPs in the *DIO1, AADAT*, and *SLC25A52* locus were smaller in BLTS, resulting in heterogeneity in the meta-analysis.

We show that a higher TSH PRS was associated with lower FT4 concentrations and a higher FT4 PRS was associated with higher TSH concentrations in our meta-analysis in childhood. This is not in line with the classic TSH stimulation model including negative feedback of T4 on the level of the hypothalamus and pituitary. Therefore, our results suggest that the identified TSH SNPs mostly act through changes in the TSH setpoint or TSH (receptor) sensitivity, while the identified FT4 SNPs are likely to act through intracellular mechanisms including sulphation, deiodination, or glucuronidation. This is in line with the limited overlap between TSH and FT4 genetic determinants observed in adults, as well as the lack of associations between FT4 SNPs and hypo- or hyperthyroidism in adults.^7^ These findings underline the importance of understanding the underlying mechanisms of individual SNPs in order to interpret their associations with clinical outcomes.

While genetic studies are less prone to confounding bias as compared to observational studies in general, inferring causality can still be hampered by possible pleiotropic effects, ie, genetic variants can influence determinants of thyroid function such as BMI and subsequently affect thyroid function. Indeed, some genetic variants of thyroid function have been associated with BMI, height, and weight circumference,^22–26^ but none of these genetic variants were associated with childhood thyroid function in the current study. In addition, the pleiotropic genetic variants might influence BMI through thyroid function instead of vice versa.

In this study, the recently discovered genetic variant of the enzyme AADAT was associated with FT4 concentrations with an effect estimate almost twice the size of that in adults.^7^ This could be caused by differential expression of *AADAT* with age, as several genes show age-dependent expression in target tissues of thyroid hormone including the brain, skin and adipose tissue.^27^ Therefore, the association between genetic variation of *AADAT* with FT4 concentrations might be more apparent at child age through higher expression of *AADAT*. To our knowledge, however, it is not known if *AADAT* expression also differs with age. *In-vitro* studies showed AADAT-dependent conversion of T4 and T3 to their metabolites. The *AADAT* variant was also associated with FT3 in our study, but was not significant anymore when including random intercepts to account for heterogeneity, which is likely explained by the different directions of effect. The *AADAT* variant was positively associated with FT3 in our meta-analysis and in ALSPAC, yet amongst the older children in BLTS, as well as in the adult GWAS there was a negative association of the *AADAT* variant with FT3.^7^ These results of an age-dependent magnitude and direction of effect might suggest that the effect of AADAT on thyroid function is age specific. However, this potential age-dependent direction of effect is based on limited data and should be considered as a hypothesis that requires further replication.

Our results seem to suggest that the effect sizes of three TSH loci, *GLIS3*, *TM4SF4*, and *VEGFA* are also notably larger in children than in adults. This difference could be related to its underlying biology related to early life rather than aging as *GLIS3* is associated with thyroid development and mutations in this gene are associated with neonatal diabetes and congenital hypothyroidism. In addition, *GLIS3* is also involved in the development of the eye, liver, kidney, and pancreatic beta cells.^28^ Similarly, TM4SF4 is a member of a protein family involved in the regulation of cell development, activation, growth and motility.^28^ Thus, the large effect of *GLIS3* and *TM4SF4* on childhood TSH concentrations might be explained by their involvement in organ and cell development, which are likely more evident during early life. VEGFA promotes conversion of T4 to T3 which consequently suppresses TSH concentrations.^29^ The large effect size of this locus can be explained by its age-dependent expression, as animal studies have shown that *VEGF* expression decreases with age.^30,31^

The loci with the largest effect sizes in childhood relative to adults have been associated with an elevated blood glucose level (*AADAT*) and diabetes (*GLIS3*). This might suggest that any relation between thyroid function and diabetes in childhood could be caused by pleiotropic genetic effects, whereas a suboptimal thyroid function in adulthood could result in long-term metabolic effects. In contrast, the loci with the smallest effect sizes in childhood relative to adults have been associated with weight (*FOXE1*) and height (*CAPZB*). This suggests that any relation between anthropometry and thyroid function in adulthood might be partially explained by pleiotropic genetic effects, whereas anthropometry might have rather direct effects on thyroid function in childhood. To our knowledge, this is the largest study on genetic determinants of childhood thyroid function. Previous studies included either small subsets of children,^9^ assessed the combined effects of a much smaller amount of currently known genetic determinants of thyroid function^7,10^ or performed a GWAS approach in adolescents in a relatively small subset of our study sample.^11^ We were able to perform this study using a multi-cohort approach with a large sample size available for analyses. This enabled us to optimize the generalizability of the results of our study by replicating adult genetic determinants and to cross-replicate our own findings. An important limitation of our study is that the design did not allow for identification of new, childhood thyroid function SNPs. We did not perform a meta-GWAS as we focused on known SNPs in adults and an even larger sample size would be preferable for this approach. Importantly, the difference in power between our study and the GWAS in adults might partially explain why we did not replicate more thyroid function SNPs. The fact that only a few of the most recently discovered TSH SNPs were replicated in children could also be a power issue, since the largest cohort was not included in this analysis and the sample size of the GWAS in which these extra TSH SNPs were identified constituted more than one and one half times the size of the previous GWAS. Future studies should include a GWAS in childhood, as new treatment targets and strategies such as the incorporation of genetics in risk stratification for treatment decisions might evolve by improving the knowledge on normal regulation of childhood thyroid function.

In conclusion, our study provides new data on genetic regulation of childhood thyroid function. Our results are evidence that the effects of many known genetic variants are already apparent in childhood. Further, we cautiously interpret our results as suggesting that the extent of the effect of certain genes may be age-specific. These findings advance the understanding of child thyroid function and aid in untangling the effects of maternal and child thyroid function on offspring outcomes.

## Supplementary Material

lvad086_Supplementary_Data

## Data Availability

The datasets generated during and/or analysed during the current study are not publicly available but may be accessed through the corresponding author on reasonable request.
