# Supplementary material for: Genetic determinants of thyroid function in children
Source: Eur J Endocrinol. 2023 Aug 2;189(2):164–74. doi: 10.1093/ejendo/lvad086 (PMC10402705; doi:10.1093/ejendo/lvad086)
Supplement: lvad086_Supplementary_Data [file lvad086_supplementary_data.pdf]

Supplemental material

Genetic determinants of thyroid function in children

Supplemental Methods.

Supplemental Table 1. Descriptive statistics of study population

Supplemental Table 2. Overview SNPs

Supplemental Table 3. TSH SNPs with TSH concentrations in separate cohorts

Supplemental Table 4. FT4 SNPs with FT4 concentrations in separate cohorts

Supplemental Table 5. TSH SNPs with TSH concentrations in cord blood

Supplemental Table 6. FT4 SNPs with FT4 concentrations in cord blood

Supplemental Table 7. Associations of TSH and FT4 PRS with thyroid function in cord blood

Supplemental Table 8. Associations of TSH unweighted PRS with thyroid function in childhood

Supplemental Table 9. Associations of FT4 unweighted PRS with thyroid function in childhood

Supplemental Table 10. FT4 SNPs with FT3 concentrations in separate cohorts

Supplemental Table 11. Meta-analysis FT4 SNPs with FT4 concentrations in the normal range of FT4

Supplemental Table 12. FT4 SNPs with FT4 concentrations in the normal range of FT4 in separate cohorts

Supplemental Table 13. Associations of different TSH PRS with TSH concentrations and different FT4 PRS with FT4 concentrations

Supplemental Table 14. Overview new TSH SNPs

Supplemental Table 15. 28 new TSH SNPs with TSH concentrations in childhood in Generation R and BLTS

Supplemental Table 16. Meta-regression of age with effect size and explained variance of FT4 PRS with FT4 concentrations

Supplemental Methods

Exclusion criteria

Children with pre-existing thyroid disease or thyroid (interfering) medication usage (levothyroxine or growth hormone) were excluded. In addition, TSH concentrations outside the cohort-specific 2.5-97.5th centile range were excluded from analyses to maintain a comparable methodology to the original study in adults, in which only subjects with TSH within this range were included for the FT4 and TSH analyses. Information on pre-existing thyroid disease and thyroid (interfering) medication was not available in BLTS. The exclusion criteria for the cord blood analyses in Generation R were maternal pre-existing thyroid disease, maternal thyroid (interfering) medication usage during pregnancy, in vitro fertilization and TSH concentrations outside the median 95% range. In addition, one child was randomly excluded from each participating sibling pair in Generation R in cord blood and child analyses.

Genetic variants and risk scores

TSH SNPs that were identified after the most recent GWAS that studied both TSH and FT4 were not included in the main analysis (1), as a comparison to only one GWAS that studied both TSH and FT4 was considered more comprehensible and consistent for this study and because the methodology of the more recent TSH GWAS was slightly different compared to the other (e.g. not excluding TSH concentrations outside cohort-specific reference range).

In Generation R, SNP data were generated using the Illumina 610 and 660W Quad platforms. MACH 1.0 software was used to impute to the 1000 Genomes phase 3 version 5 reference panel. In ALSPAC, SNP data were obtained with the Illumina HumanHap550 quad chip genotyping platforms and subsequent imputation to the 1000 Genomes phase 1 version 3 reference panel using Impute V2.2.2. Three proxies (rs116552240, rs143841293, rs199437 with r^2 >0.99) were selected for the TSH SNPs (rs8176645, rs1265091, and rs77819282) that were not available. In BLTS,

SNP data were obtained using either the HumanCoreExome-12v1.0 or IlluminaHuman610WQuad bead chip and subsequent imputation to the Haplotype Reference Consortium r1.1 reference panel. Perfect proxies (rs10838757, rs11801304, and rs78676901 with r^2 =1) were selected for one FT4 SNP (rs11039355) and two TSH SNPs (rs74804879 and rs182873197) which were not available. No proxy was available for one TSH indel (rs200574439) in any cohort. Information on stringent quality control has been described in detail elsewhere for Generation R (2), ALSPAC (3), and BLTS (4).

Supplemental Table 1. Descriptive statistics of study population

Characteristic	Generation R Cord blood N	Generation R Cord blood **Median**	Generation R Cord blood (95% range)[a]	Generation R Childhood N	Generation R Childhood **Median**	Generation R Childhood (95% range)[a]	ALSPAC N	ALSPAC **Median**	ALSPAC (95% range)[a]	BLTS N	BLTS **Median**	BLTS (95% range)[a]
**Age at blood sampling,** years	NA			2169	6.0	(5.7-7.5)	3382	7.5	(7.3-8.9)	1680	12.1	(12.0-12.5)
**Gestational age at blood sampling,** weeks	2388	40.3	(36.7-42.4)	NA			NA			NA		
**Female sex**, %	1178	49.3		1068	49.2		1625	48.0		851	50.7	
**TSH**, mU/L	2388	9.7	(4.0-27.2)[b]	2169	2.4	(1.1-4.7)[b]	3382	2.1	(1.1-4.1)[b]	1680	1.5	(0.8-2.8)
**FT4**, pmol/L	2374	20.5	(15.6-28.2)	2159	16.5	(13.7-20.3)	3367	15.6	(12.8-19.3)	1680	12.6	(10.7-14.9)
**FT3**, pmol/L	NA			NA			3343	6.3	(5.1-7.6)	1680	5.3	(4.6-6.1)

[a]2.5[th] and 97.5[th] percentile
[b]The 2.5[th]-97.5[th] percentile range after selecting on TSH cohort-specific reference ranges. TSH 2.5[th]-97.5[th] percentile ranges used for selecting TSH cohort-specific reference range: GenR cord blood (3.3-32.9), GenR childhood (0.9-5.2), ALSPAC (0.9-4.8)

Supplemental Table 2. Overview SNPs

TSH SNPs

SNP	Chr	Position	Locus	A1	A2	AF1 GenR wave 1 cord blood	AF1 GenR wave 2 cord blood	AF1 GenR wave 1 child	AF1 GenR wave 2 child	AF1 ALSPAC	AF1 BLTS
rs12089835	1	19771438	CAPZB	t	c	0.34	0.32	0.34	0.34	0.36	0.37
rs10917469	1	19843576	CAPZB	a	g	0.84	0.85	0.84	0.85	0.85	0.85
rs74804879*	1	19862320	CAPZB	t	c	0.62	0.63	0.62	0.65	0.66	0.74
rs334725	1	61610049	NFIA	a	g	0.95	0.91	0.95	0.94	0.96	0.95
rs17020122	1	108357391	VAV3	t	c	0.09	0.10	0.10	0.09	0.09	0.09
rs16856540	2	217580413	IGFBP5	t	c	0.85	0.87	0.84	0.86	0.85	0.85
rs13015993	2	217625523	IGFBP5	a	g	0.72	0.70	0.74	0.73	0.75	0.75
rs6724073	2	218236786	DIRC3	t	c	0.74	0.70	0.74	0.74	0.74	0.76
rs1663070	3	12239852	SYN2	t	c	0.74	0.73	0.75	0.74	0.74	0.75
rs28502438	3	149220109	TM4SF4	t	c	0.58	0.58	0.58	0.55	0.58	0.56
rs13100823	3	185514088	IGF2BP2	t	c	0.29	0.24	0.28	0.28	0.30	0.33
rs59381142	3	193916181	HES1	a	g	0.24	0.24	0.24	0.23	0.25	0.25
rs6535624	4	149587905	NR3C2	a	g	0.43	0.39	0.42	0.43	0.43	0.42
rs11732089	4	149665602	NR3C2	t	c	0.80	0.80	0.80	0.79	0.79	0.80
rs62362610	5	76439961	PDE8B	c	g	0.08	0.10	0.08	0.09	0.10	0.08
rs1119208	5	76488613	PDE8B	t	c	0.34	0.37	0.35	0.36	0.34	0.34
rs139424329	5	76495539	PDE8B	a	g	0.02	0.01	0.01	0.01	0.01	0.01
rs2127387	5	76532571	PDE8B	a	g	0.39	0.41	0.39	0.41	0.40	0.40
rs7702192	5	76554807	PDE8B	a	c	0.49	0.49	0.47	0.49	0.48	0.47
rs113974964	5	76652403	PDE8B	t	c	0.04	0.03	0.04	0.04	0.04	0.04
rs139149784	5	76660193	PDE8B	a	g	0.01	0.01	0.01	0.01	0.02	0.01
rs182873197*	5	76773148	PDE8B	t	c	0.06	0.03	0.06	0.06	0.05	0.05
rs1265091*	6	31108129	PSORS1C1	t	c	0.15	0.12	0.15	0.15	0.19*	0.17
rs744103	6	43805362	VEGFA/LOC100132354	a	t	0.68	0.66	0.68	0.67	0.67	0.70
rs9381266	6	43905037	VEGFA/LOC100132354	t	c	0.75	0.76	0.76	0.76	0.75	0.76
rs9497965	6	148521292	SASH1	t	c	0.40	0.41	0.41	0.40	0.41	0.39
rs73022105	6	165973757	PDE10A	t	c	0.96	0.95	0.96	0.95	0.96	0.95
rs1079418	6	166047034	PDE10A	a	g	0.68	0.65	0.67	0.68	0.68	0.70
rs56009477	8	23356964	SLC25A37	a	g	0.87	0.86	0.87	0.87	0.84	0.84
rs2439301	8	32433013	NRG1	a	g	0.23	0.24	0.23	0.24	0.25	0.25

rs10957494	8	70365025	SULF1	a	g	0.70	0.73	0.71	0.73	0.71	0.69
rs118039499	8	133771635	TG	a	c	0.98	0.99	0.98	0.98	0.97	0.98
rs2739067	8	133951991	TG	a	g	0.62	0.61	0.61	0.61	0.62	0.62
rs10814915	9	4290544	GLIS3	t	c	0.44	0.49	0.44	0.48	0.41	0.44
rs9298749	9	16214340	C9orf92	a	c	0.62	0.61	0.60	0.63	0.62	0.62
rs8176645*	9	136149098	ABO	a	t	0.35	0.36	0.34	0.36	0.35*	0.30
rs11255790	10	8682180	GATA3	t	c	0.30	0.26	0.30	0.30	0.30	0.32
rs4933466	10	89849519	PTEN	a	g	0.60	0.60	0.61	0.58	0.59	0.59
rs12284404	11	45228686	PRDM11	a	g	0.27	0.31	0.28	0.29	0.29	0.28
rs4445669	11	115045237	CADM1	t	c	0.46	0.50	0.46	0.46	0.44	0.46
rs7329958	13	24782080	SPATA13	t	c	0.34	0.30	0.34	0.34	0.34	0.33
rs398745	14	36536181	MBIP	a	c	0.59	0.62	0.60	0.64	0.59	0.58
rs2254613	14	36713154	MBIP	t	g	0.56	0.57	0.57	0.58	0.52	0.53
rs11159482	14	81490842	TSHR	t	c	0.06	0.08	0.06	0.08	0.07	0.18
rs59334515	14	81594143	TSHR	t	c	0.23	0.22	0.23	0.22	0.23	0.24
rs12893151	14	81619945	TSHR	a	c	0.21	0.22	0.21	0.18	0.20	0.21
rs8015085	14	93585331	ITPK1	a	g	0.20	0.15	0.18	0.18	0.20	0.19
rs17477923	15	49711185	FAM227B/FGF7	t	c	0.72	0.75	0.73	0.74	0.76	0.73
rs11639111	15	49749735	FAM227B/FGF7	t	c	0.38	0.40	0.38	0.34	0.42	0.38
rs13329353	15	89113877	DET1	t	c	0.69	0.70	0.69	0.71	0.67	0.67
rs1045476	16	4015313	ADCY9	a	g	0.19	0.15	0.19	0.17	0.18	0.18
rs30227	16	14405428	MIR365A	t	c	0.59	0.54	0.59	0.62	0.60	0.61
rs17767491	16	79745487	MAF	a	g	0.69	0.70	0.70	0.70	0.69	0.69
rs77819282*	17	44762589	NSF	a	g	0.24	0.25	0.25	0.23	0.26*	0.26
rs1157994	17	59338574	BCAS3	a	g	0.04	0.08	0.04	0.07	0.04	0.04
rs1042673	17	70121339	SOX9	a	g	0.53	0.55	0.52	0.52	0.53	0.50
rs963384	17	70369758	SOX9	t	c	0.46	0.53	0.47	0.49	0.48	0.47
rs4804413	19	7222655	INSR	t	c	0.44	0.42	0.44	0.42	0.42	0.42
rs1203944	20	22596879	FOXA2	t	c	0.21	0.23	0.22	0.22	0.22	0.21
rs12390237	23	3612081	PRKX	a	g	0.72	0.67	0.72	0.63	0.62	0.72

FT4 SNPs

SNP	Chr	Position	Locus	A1	A2	AF1 GenR wave 1 cord blood	AF1 GenR wave 2 cord blood	AF1 GenR wave 1 child	AF1 GenR wave 2 child	AF1 ALSPAC	AF1 BLTS
rs145019385	1	54252139	DIO1	t	c	0.98	0.99	0.98	0.98	0.97	0.98
rs12033572	1	54369674	DIO1	c	g	0.03	0.02	0.03	0.02	0.04	0.05
rs2235544	1	54375570	DIO1	a	c	0.54	0.53	0.53	0.54	0.52	0.53
rs954878	1	54578401	DIO1	a	g	0.43	0.40	0.42	0.39	0.45	0.45
rs4954192	2	135632981	ACMSD	t	c	0.44	0.42	0.43	0.42	0.37	0.41
rs6785807	3	181718601	SOX2-OT	a	g	0.15	0.18	0.14	0.16	0.16	0.18
rs6854291	4	170992760	AADAT	a	g	0.10	0.12	0.09	0.10	0.09	0.10
rs10946313	6	19381386	ID4	t	c	0.63	0.62	0.62	0.63	0.63	0.64
rs9356988	6	25777481	SLC17A4	a	g	0.26	0.24	0.26	0.26	0.27	0.26
rs137964359	6	26001742	SLC17A4	t	c	0.99	0.99	0.99	0.99	0.98	0.99
rs17185536	6	100620931	LOC728012	t	c	0.22	0.24	0.23	0.22	0.24	0.24
rs67583169	8	61212179	CA8	c	g	0.87	0.83	0.87	0.86	0.87	0.86
rs10119187	9	4223660	GLIS3	t	c	0.82	0.79	0.82	0.82	0.84	0.82
rs10739496	9	100552559	FOXE1	t	c	0.68	0.67	0.68	0.68	0.67	0.68
rs10984606	9	100739117	FOXE1	t	g	0.48	0.48	0.48	0.50	0.47	0.49
rs10818937	9	127015440	NEK6	t	c	0.31	0.32	0.32	0.29	0.31	0.33
rs4842131	9	139092679	LHX3	t	c	0.43	0.42	0.43	0.46	0.42	0.44
rs55679545	9	139122363	LHX3	a	g	0.24	0.21	0.24	0.25	0.26	0.28
rs11039355*	11	47737501	FNBP4	t	c	0.34	0.35	0.34	0.32	0.34	0.36
rs4149056	12	21331549	SLCO1B1	t	c	0.85	0.78	0.85	0.83	0.85	0.86
rs150816132	14	80464293	DIO2	a	g	0.01	0.01	0.01	0.01	0.01	0.01
rs978055	14	80534869	DIO2	a	t	0.38	0.37	0.37	0.37	0.39	0.38
rs225014	14	80669580	DIO2	t	c	0.64	0.70	0.63	0.64	0.64	0.64
rs12323871	14	101852075	DIO3OS	t	c	0.81	0.81	0.81	0.82	0.82	0.83
rs11626434	14	101998443	DIO3OS	c	g	0.36	0.34	0.36	0.34	0.35	0.36
rs12907106	15	63873658	USP3	c	g	0.26	0.24	0.26	0.28	0.27	0.30
rs8063103	16	12703395	SNX29	c	g	0.85	0.88	0.86	0.85	0.85	0.86
rs11078333	17	16049626	NCOR1	a	t	0.47	0.50	0.46	0.48	0.51	0.48
rs1080094	18	29173795	SLC25A52	a	g	0.59	0.58	0.60	0.60	0.61	0.61
rs113107469	18	29306737	SLC25A52	t	c	0.04	0.03	0.04	0.04	0.04	0.04
rs56069042	18	57914644	MC4R	a	g	0.96	0.98	0.95	0.96	0.96	0.97

*Proxies:

rs11801304 is a proxy for rs74804879 in BLTS

rs78676901 is a proxy for rs182873197 in BLTS

rs143841293 is a proxy for rs1265091 in ALSPAC

rs116552240 is a proxy for rs8176645 in ALSPAC

rs199437 is a proxy for rs77819282 in ALSPAC
rs10838757 is a proxy for rs11039355 in BLTS

Abbreviations: SNP= Single Nucleotide Polymorphism, Chr= Chromosome, A1= Allele 1, A2= Allele 2, AF1= Allele frequency of allele 1

Supplemental Table 3. TSH SNPs with TSH concentrations in separate cohorts

							GenR wave 1				GenR wave 2				ALSPAC				BLTS			
SNP	Chr	Position	Locus	A1	A2	b Adults	b	se	pval	p.fdr	b	se	pval	p.fdr	b	se	pval	p.fdr	b	se	pval	p.fdr
rs12089835	1	19771438	CAPZB	t	c	0.073	-0.050	0.038	0.19	0.43	0.001	0.062	0.98	>0.99	-0.025	0.026	0.33	0.52	-0.008	0.038	0.84	0.92
rs10917469	1	19843576	CAPZB	a	g	0.111	-0.038	0.049	0.44	0.67	0.059	0.077	0.44	0.67	-0.010	0.034	0.77	0.84	0.032	0.051	0.53	0.74
rs74804879	1	19862320	CAPZB	t	c	0.05	-0.010	0.037	0.78	0.92	0.051	0.063	0.42	0.67	0.022	0.026	0.41	0.59	0.024	0.042	0.56	0.74
rs334725	1	61610049	NFIA	a	g	0.174	-0.010	0.083	0.90	0.95	0.178	0.155	0.25	0.58	0.090	0.059	0.13	0.27	0.253	0.086	<0.01	0.03
rs17020122	1	108357391	VAV3	t	c	0.104	0.069	0.059	0.24	0.52	0.150	0.096	0.12	0.43	0.145	0.042	<0.01	0.00	0.225	0.065	<0.01	0.03
rs16856540	2	217580413	IGFBP5	t	c	-0.055	-0.040	0.050	0.42	0.67	0.102	0.083	0.22	0.58	-0.067	0.034	<0.05	0.14	-0.029	0.051	0.57	0.74
rs13015993	2	217625523	IGFBP5	a	g	0.082	0.077	0.042	0.06	0.21	0.114	0.064	0.08	0.33	0.130	0.028	<0.001	0.00	0.121	0.041	<0.01	0.03
rs6724073	2	218236786	DIRC3	t	c	0.045	0.028	0.047	0.55	0.74	0.033	0.074	0.65	0.81	0.114	0.031	<0.001	0.00	-0.039	0.041	0.34	0.59
rs1663070	3	12239852	SYN2	t	c	-0.046	-0.088	0.041	0.03	0.16	-0.082	0.065	0.21	0.58	-0.071	0.027	0.01	0.04	0.006	0.040	0.88	0.93
rs28502438	3	149220109	TM4SF4	t	c	0.035	0.027	0.036	0.45	0.68	0.070	0.061	0.25	0.58	0.056	0.024	0.02	0.07	0.107	0.037	<0.01	0.03
rs13100823	3	185514088	IGF2BP2	t	c	-0.042	-0.072	0.041	0.08	0.22	-0.096	0.062	0.12	0.43	0.011	0.027	0.69	0.79	-0.061	0.039	0.12	0.27
rs59381142	3	193916181	HES1	a	g	-0.054	-0.047	0.044	0.29	0.56	-0.131	0.071	0.07	0.33	-0.062	0.029	0.03	0.10	-0.063	0.040	0.12	0.27
rs6535624	4	149587905	NR3C2	a	g	0.042	0.066	0.037	0.07	0.21	0.043	0.058	0.46	0.67	0.072	0.024	<0.01	0.02	-0.021	0.038	0.57	0.74
rs11732089	4	149665602	NR3C2	t	c	0.115	0.236	0.045	<0.001	<0.001	0.243	0.069	<0.001	0.01	0.213	0.029	<0.001	0.00	0.123	0.045	0.01	0.05
rs62362610	5	76439961	PDE8B	c	g	0.073	-0.043	0.068	0.53	0.73	-0.025	0.106	0.81	0.90	0.008	0.043	0.85	0.90	-0.059	0.067	0.38	0.60
rs1119208	5	76488613	PDE8B	t	c	0.046	0.038	0.039	0.33	0.59	0.064	0.061	0.30	0.66	0.023	0.026	0.38	0.58	0.081	0.038	0.04	0.10
rs139424329	5	76495539	PDE8B	a	g	-0.200	-0.027	0.158	0.87	0.93	-0.178	0.288	0.54	0.77	-0.006	0.153	0.97	0.99	0.054	0.180	0.77	0.88
rs2127387	5	76532571	PDE8B	a	g	0.144	0.140	0.037	<0.001	<0.01	0.194	0.061	<0.01	0.03	0.143	0.025	<0.001	0.00	0.061	0.036	0.10	0.23
rs7702192	5	76554807	PDE8B	a	c	0.07	0.091	0.035	0.01	0.09	0.044	0.057	0.45	0.67	0.065	0.024	0.01	0.03	0.054	0.037	0.14	0.28
rs113974964	5	76652403	PDE8B	t	c	-0.124	-0.171	0.091	0.06	0.21	-0.035	0.140	0.80	0.90	-0.123	0.063	0.05	0.14	-0.224	0.094	0.02	0.06
rs139149784	5	76660193	PDE8B	a	g	0.156	0.543	0.201	0.01	0.07	0.132	0.240	0.58	0.78	0.076	0.113	0.50	0.65	-0.038	0.156	0.81	0.90
rs182873197	5	76773148	PDE8B	t	c	-0.08	-0.119	0.075	0.11	0.30	0.071	0.120	0.56	0.78	-0.024	0.060	0.69	0.79	0.013	0.087	0.88	0.93
rs1265091	6	31108129	PSORS1C1	t	c	0.058	0.110	0.053	0.04	0.17	0.098	0.078	0.21	0.58	0.067	0.031	0.03	0.10	-0.035	0.049	0.48	0.71
rs744103	6	43805362	VEGFA/ LOC100132354	a	t	0.092	0.089	0.040	0.03	0.16	0.159	0.064	0.01	0.17	0.106	0.027	<0.001	0.00	0.095	0.038	0.01	0.05
rs9381266	6	43905037	VEGFA/ LOC100132354	t	c	0.073	0.157	0.042	<0.001	<0.01	0.207	0.065	<0.01	0.03	0.111	0.028	<0.001	0.00	0.104	0.042	0.01	0.05
rs9497965	6	148521292	SASH1	t	c	0.044	0.088	0.038	0.02	0.13	-0.017	0.057	0.77	0.89	0.014	0.025	0.59	0.73	0.100	0.038	0.01	0.05
rs73022105	6	165973757	PDE10A	t	c	0.105	0.188	0.092	0.04	0.17	0.134	0.161	0.41	0.67	0.023	0.061	0.70	0.79	0.097	0.082	0.24	0.45
rs1079418	6	166047034	PDE10A	a	g	0.101	0.239	0.038	<0.001	<0.001	0.233	0.061	<0.001	0.01	0.120	0.026	<0.001	0.00	0.082	0.040	0.04	0.11
rs56009477	8	23356964	SLC25A37	a	g	0.050	0.014	0.052	0.79	0.92	0.000	0.085	>0.99	>0.99	0.039	0.033	0.24	0.43	0.045	0.050	0.36	0.59

rs2439301	8	32433013	NRG1	a	g	-0.059	-0.091	0.044	0.04	0.17	-0.080	0.070	0.25	0.58	-0.051	0.029	0.08	0.20	-0.032	0.042	0.44	0.67
rs10957494	8	70365025	SULF1	a	g	-0.036	0.003	0.040	0.94	0.95	-0.028	0.065	0.67	0.81	0.000	0.027	0.99	0.99	-0.001	0.040	0.98	0.98
rs118039499	8	133771635	TG	a	c	0.185	0.140	0.138	0.31	0.58	0.170	0.227	0.45	0.67	0.227	0.079	<0.01	0.02	0.303	0.116	0.01	0.05
rs2739067	8	133951991	TG	a	g	-0.042	-0.003	0.037	0.93	0.95	-0.087	0.059	0.14	0.45	-0.024	0.025	0.33	0.52	-0.056	0.037	0.13	0.27
rs10814915	9	4290544	GLIS3	t	c	0.042	0.108	0.036	<0.01	0.04	0.107	0.057	0.06	0.33	0.048	0.025	0.05	0.14	0.097	0.036	0.01	0.05
rs9298749	9	16214340	C9orf92	a	c	-0.038	-0.051	0.038	0.18	0.43	-0.013	0.063	0.84	0.92	0.012	0.026	0.64	0.76	0.047	0.039	0.23	0.44
rs8176645	9	136149098	ABO	a	t	0.052	0.063	0.038	0.10	0.27	0.054	0.058	0.36	0.67	0.043	0.025	0.09	0.21	0.124	0.040	<0.01	0.03
rs11255790	10	8682180	GATA3	t	c	-0.039	-0.044	0.040	0.27	0.54	-0.063	0.063	0.32	0.67	0.015	0.027	0.56	0.72	-0.064	0.039	0.10	0.23
rs4933466	10	89849519	PTEN	a	g	0.037	0.025	0.037	0.49	0.71	0.132	0.062	0.03	0.28	0.033	0.025	0.17	0.34	0.010	0.036	0.78	0.88
rs12284404	11	45228686	PRDM11	a	g	-0.067	-0.047	0.039	0.23	0.50	0.001	0.060	0.99	>0.99	-0.065	0.027	0.02	0.06	-0.036	0.041	0.38	0.60
rs4445669	11	115045237	CADM1	t	c	-0.039	-0.036	0.037	0.33	0.59	0.045	0.058	0.43	0.67	-0.025	0.025	0.32	0.52	-0.111	0.036	<0.01	0.03
rs7329958	13	24782080	SPATA13	t	c	-0.044	-0.034	0.039	0.39	0.63	-0.054	0.065	0.41	0.67	0.033	0.026	0.20	0.38	-0.016	0.039	0.69	0.82
rs398745	14	36536181	MBIP	a	c	-0.052	0.011	0.036	0.76	0.92	-0.035	0.064	0.59	0.78	-0.072	0.024	<0.01	0.02	0.015	0.037	0.68	0.82
rs2254613	14	36713154	MBIP	t	g	-0.035	0.007	0.039	0.86	0.93	-0.032	0.064	0.61	0.79	-0.067	0.027	0.01	0.05	-0.022	0.036	0.56	0.74
rs11159482	14	81490842	TSHR	t	c	0.085	-0.077	0.084	0.36	0.62	0.212	0.119	0.08	0.33	0.087	0.053	0.10	0.23	0.172	0.069	0.01	0.05
rs59334515	14	81594143	TSHR	t	c	-0.054	-0.101	0.043	0.02	0.13	-0.084	0.069	0.22	0.58	-0.011	0.029	0.71	0.79	-0.053	0.043	0.22	0.43
rs12893151	14	81619945	TSHR	a	c	-0.057	0.022	0.048	0.65	0.83	-0.134	0.078	0.09	0.35	-0.025	0.032	0.43	0.61	-0.018	0.044	0.68	0.82
rs8015085	14	93585331	ITPK1	a	g	0.067	0.113	0.052	0.03	0.16	0.175	0.078	0.02	0.24	0.024	0.031	0.44	0.61	0.106	0.045	0.02	0.06
rs17477923	15	49711185	FAM227B/ FGF7	t	c	0.083	0.017	0.041	0.67	0.83	0.023	0.064	0.72	0.84	-0.014	0.028	0.63	0.76	0.105	0.040	0.01	0.05
rs11639111	15	49749735	FAM227B/ FGF7	t	c	0.045	0.002	0.037	0.95	0.95	-0.010	0.067	0.89	0.95	0.005	0.025	0.84	0.90	0.019	0.037	0.61	0.78
rs13329353	15	89113877	DET1	t	c	0.061	0.009	0.040	0.82	0.92	0.023	0.062	0.71	0.84	0.017	0.026	0.50	0.65	0.090	0.038	0.02	0.06
rs1045476	16	4015313	ADCY9	a	g	0.047	0.026	0.050	0.61	0.80	0.040	0.081	0.62	0.79	0.059	0.032	0.07	0.18	-0.112	0.048	0.02	0.06
rs30227	16	14405428	MIR365A	t	c	-0.046	-0.024	0.039	0.54	0.74	0.005	0.060	0.94	0.99	-0.001	0.025	0.96	0.99	-0.036	0.036	0.32	0.57
rs17767491	16	79745487	MAF	a	g	0.088	0.069	0.038	0.07	0.21	0.062	0.061	0.31	0.67	0.131	0.026	<0.001	0.00	0.116	0.039	<0.01	0.03
rs77819282	17	44762589	NSF	a	g	0.043	-0.009	0.043	0.84	0.93	0.150	0.072	0.04	0.28	0.027	0.028	0.34	0.52	0.001	0.042	0.98	0.98
rs1157994	17	59338574	BCAS3	a	g	-0.083	-0.067	0.099	0.50	0.71	-0.188	0.128	0.14	0.45	-0.097	0.061	0.11	0.25	0.007	0.086	0.94	0.97
rs1042673	17	70121339	SOX9	a	g	-0.055	0.009	0.036	0.81	0.92	0.046	0.058	0.43	0.67	-0.029	0.025	0.25	0.45	-0.035	0.038	0.36	0.59
rs963384	17	70369758	SOX9	t	c	0.035	0.041	0.037	0.26	0.53	0.047	0.059	0.43	0.67	0.027	0.025	0.27	0.46	0.061	0.037	0.10	0.23
rs4804413	19	7222655	INSR	t	c	0.053	0.032	0.035	0.37	0.62	-0.057	0.060	0.34	0.67	0.018	0.025	0.46	0.63	0.091	0.036	0.01	0.05
rs1203944	20	22596879	FOXA2	t	c	-0.051	-0.080	0.043	0.07	0.21	-0.141	0.070	0.04	0.28	-0.041	0.030	0.16	0.33	-0.108	0.045	0.02	0.06
rs12390237	23	3612081	PRKX	a	g	-0.046	-0.076	0.051	0.14	0.35	0.101	0.051	<0.05	0.28	-0.033	0.024	0.16	0.33	-0.014	0.044	0.75	0.88

Supplemental Table 4. FT4 SNPs with FT4 concentrations in separate cohorts

							GenR wave 1				GenR wave 2				ALSPAC				BLTS			
SNP	Chr	Position	Locus	A1	A2	b Adults	b	se	pval	p.fdr	b	se	pval	p.fdr	b	se	pval	p.fdr	b	se	pval	p.fdr
rs145019385	1	54252139	DIO1	t	c	0.181	0.199	0.164	0.23	0.39	-0.117	0.238	0.62	0.71	0.095	0.084	0.26	0.44	-0.119	0.120	0.32	0.52
rs12033572	1	54369674	DIO1	c	g	0.115	0.317	0.109	<0.01	0.04	0.143	0.231	0.53	0.71	0.088	0.061	0.15	0.36	0.124	0.086	0.15	0.39
rs2235544	1	54375570	DIO1	a	c	0.139	0.255	0.035	<0.001	<0.001	0.138	0.057	0.02	0.10	0.183	0.024	<0.001	<0.001	0.097	0.035	0.01	0.19
rs954878	1	54578401	DIO1	a	g	-0.058	-0.008	0.036	0.82	0.90	-0.097	0.061	0.11	0.34	-0.080	0.024	<0.01	0.01	0.052	0.036	0.15	0.39
rs4954192	2	1.36E+08	ACMSD	t	c	-0.033	-0.072	0.040	0.08	0.26	0.056	0.060	0.35	0.64	-0.044	0.025	0.08	0.22	0.006	0.035	0.85	0.91
rs6785807	3	1.82E+08	SOX2-OT	a	g	-0.057	-0.051	0.053	0.34	0.55	-0.139	0.076	0.07	0.30	-0.040	0.033	0.22	0.44	-0.018	0.047	0.69	0.83
rs6854291	4	1.71E+08	AADAT	a	g	0.117	0.271	0.063	<0.001	<0.001	0.459	0.098	<0.001	<0.001	0.214	0.043	<0.001	<0.001	0.147	0.060	0.01	0.20
rs10946313	6	19381386	ID4	t	c	0.044	0.024	0.037	0.52	0.77	0.028	0.060	0.64	0.71	0.018	0.025	0.48	0.65	-0.034	0.036	0.34	0.52
rs9356988	6	25777481	SLC17A4	a	g	-0.052	-0.062	0.040	0.12	0.35	-0.063	0.067	0.34	0.64	-0.018	0.028	0.50	0.65	-0.005	0.040	0.90	0.93
rs137964359	6	26001742	SLC17A4	t	c	-0.200	-0.045	0.266	0.87	0.93	-0.509	0.961	0.60	0.71	-0.075	0.097	0.44	0.62	-0.366	0.164	0.03	0.20
rs17185536	6	1.01E+08	LOC728012	t	c	0.071	0.003	0.049	0.94	0.94	-0.009	0.080	0.91	0.91	0.079	0.030	0.01	0.03	-0.008	0.042	0.84	0.91
rs67583169	8	61212179	CA8	c	g	0.062	0.074	0.055	0.18	0.39	-0.102	0.084	0.23	0.50	0.013	0.036	0.72	0.79	0.034	0.051	0.50	0.70
rs10119187	9	4223660	GLIS3	t	c	0.048	-0.043	0.047	0.37	0.57	0.150	0.079	0.06	0.29	-0.021	0.033	0.53	0.65	-0.004	0.047	0.94	0.94
rs10739496	9	1.01E+08	FOXE1	t	c	0.078	0.049	0.038	0.20	0.39	0.025	0.062	0.69	0.74	0.054	0.026	0.04	0.12	0.021	0.039	0.60	0.77
rs10984606	9	1.01E+08	FOXE1	t	g	-0.04	-0.013	0.036	0.71	0.85	0.031	0.058	0.60	0.71	0.020	0.024	0.40	0.62	-0.007	0.035	0.85	0.91
rs10818937	9	1.27E+08	NEK6	t	c	-0.039	-0.012	0.039	0.76	0.87	-0.080	0.064	0.22	0.50	0.005	0.026	0.84	0.90	-0.064	0.039	0.10	0.36
rs4842131	9	1.39E+08	LHX3	t	c	-0.104	-0.094	0.040	0.02	0.11	-0.057	0.059	0.34	0.64	-0.071	0.026	0.01	0.03	-0.052	0.035	0.14	0.39
rs55679545	9	1.39E+08	LHX3	a	g	0.044	-0.065	0.053	0.22	0.39	0.051	0.068	0.46	0.71	0.015	0.029	0.62	0.74	0.038	0.038	0.32	0.52
rs11039355	11	47737501	FNBP4	t	c	-0.039	0.016	0.038	0.67	0.83	-0.045	0.065	0.49	0.71	-0.039	0.026	0.14	0.36	-0.040	0.037	0.28	0.52
rs4149056	12	21331549	SLCO1B1	t	c	-0.048	-0.028	0.049	0.57	0.77	-0.215	0.074	<0.01	0.06	-0.041	0.035	0.24	0.44	0.051	0.049	0.30	0.52
rs150816132	14	80464293	DIO2	a	g	-0.22	-0.379	0.185	0.04	0.18	-0.452	0.292	0.12	0.35	-0.384	0.134	<0.01	0.02	-0.198	0.154	0.20	0.45
rs978055	14	80534869	DIO2	a	t	0.038	0.061	0.038	0.11	0.33	0.044	0.061	0.47	0.71	-0.033	0.025	0.19	0.41	0.062	0.036	0.09	0.36
rs225014	14	80669580	DIO2	t	c	0.047	0.047	0.038	0.22	0.39	0.078	0.060	0.20	0.50	0.001	0.025	0.98	0.98	0.041	0.037	0.27	0.52
rs12323871	14	1.02E+08	DIO3OS	t	c	-0.047	-0.060	0.046	0.19	0.39	0.144	0.090	0.11	0.34	-0.066	0.031	0.04	0.12	-0.086	0.047	0.07	0.35
rs11626434	14	1.02E+08	DIO3OS	c	g	0.053	0.102	0.038	0.01	0.06	0.172	0.063	0.01	0.06	0.073	0.026	0.01	0.02	0.086	0.037	0.02	0.20
rs12907106	15	63873658	USP3	c	g	-0.039	0.005	0.041	0.90	0.93	-0.016	0.066	0.81	0.84	-0.021	0.027	0.43	0.62	-0.016	0.039	0.68	0.83
rs8063103	16	12703395	SNX29	c	g	-0.051	-0.075	0.053	0.16	0.39	-0.144	0.088	0.10	0.34	0.002	0.035	0.95	0.98	-0.034	0.052	0.52	0.70
rs11078333	17	16049626	NCOR1	a	t	0.042	0.082	0.036	0.02	0.11	0.045	0.063	0.48	0.71	0.027	0.025	0.27	0.44	0.069	0.036	0.05	0.33
rs1080094	18	29173795	SLC25A52	a	g	-0.042	0.021	0.037	0.57	0.77	0.027	0.059	0.64	0.71	0.011	0.025	0.66	0.76	-0.045	0.036	0.20	0.45
rs113107469	18	29306737	SLC25A52	t	c	0.200	-0.045	0.100	0.66	0.83	-0.107	0.163	0.51	0.71	0.339	0.066	<0.001	<0.001	0.078	0.095	0.41	0.61
rs56069042	18	57914644	MC4R	a	g	0.099	0.175	0.090	0.05	0.20	0.373	0.141	0.01	0.06	0.079	0.070	0.26	0.44	0.166	0.102	0.10	0.36

Supplemental Table 5. TSH SNPs with TSH concentrations in cord blood

							Meta-analysis GenR wave 1 and 2					GenR wave 1				GenR wave 2			
SNP	Chr	Position	Locus	A1	A2	b Adults	b	se	pval	p.fdr	I2	b	se	pval	p.fdr	b	se	pval	p.fdr
rs12089835	1	19771438	CAPZB	t	c	0.073	-0.068	0.031	0.03	0.19	0	-0.067	0.031	0.03	0.18	-0.115	0.170	0.50	0.95
rs10917469	1	19843576	CAPZB	a	g	0.111	0.042	0.040	0.30	0.68	0	0.049	0.041	0.23	0.53	-0.165	0.212	0.44	0.95
rs74804879	1	19862320	CAPZB	t	c	0.050	-0.011	0.030	0.71	0.90	0	-0.011	0.031	0.72	0.89	-0.009	0.145	0.95	0.95
rs334725	1	61610049	NFIA	a	g	0.174	0.045	0.065	0.48	0.81	0	0.039	0.066	0.56	0.79	0.162	0.285	0.57	0.95
rs17020122	1	108357391	VAV3	t	c	0.104	0.057	0.049	0.25	0.65	14	0.045	0.051	0.37	0.75	0.291	0.223	0.20	0.95
rs16856540	2	217580413	IGFBP5	t	c	-0.055	-0.027	0.041	0.51	0.81	0	-0.023	0.042	0.58	0.79	-0.126	0.208	0.55	0.95
rs13015993	2	217625523	IGFBP5	a	g	0.082	0.002	0.033	0.96	0.97	16	-0.006	0.033	0.87	0.97	0.174	0.161	0.28	0.95
rs6724073	2	218236786	DIRC3	t	c	0.045	0.026	0.038	0.50	0.81	0	0.024	0.039	0.54	0.79	0.064	0.178	0.72	0.95
rs1663070	3	12239852	SYN2	t	c	-0.046	-0.002	0.033	0.94	0.97	0	0.003	0.034	0.93	0.97	-0.114	0.155	0.46	0.95
rs28502438	3	149220109	TM4SF4	t	c	0.035	0.102	0.029	<0.001	0.01	0	0.099	0.030	<0.01	0.03	0.193	0.145	0.19	0.95
rs13100823	3	185514088	IGF2BP2	t	c	-0.042	-0.069	0.032	0.03	0.19	0	-0.068	0.032	0.04	0.18	-0.108	0.167	0.52	0.95
rs59381142	3	193916181	HES1	a	g	-0.054	-0.004	0.036	0.91	0.97	0	0.000	0.036	>0.99	>0.99	-0.102	0.184	0.58	0.95
rs6535624	4	149587905	NR3C2	a	g	0.042	0.003	0.029	0.93	0.97	46	0.011	0.030	0.72	0.89	-0.196	0.148	0.19	0.95
rs11732089	4	149665602	NR3C2	t	c	0.115	0.008	0.037	0.84	0.97	43	0.017	0.037	0.65	0.86	-0.233	0.186	0.21	0.95
rs62362610	5	76439961	PDE8B	c	g	0.073	0.076	0.055	0.17	0.55	0	0.077	0.056	0.17	0.50	0.061	0.238	0.80	0.95
rs1119208	5	76488613	PDE8B	t	c	0.046	0.091	0.031	<0.01	0.04	0	0.088	0.032	0.01	0.05	0.148	0.161	0.36	0.95
rs139424329	5	76495539	PDE8B	a	g	-0.200	-0.203	0.121	0.10	0.44	0	-0.203	0.121	0.10	0.41	1.881	26.910	0.94	0.95
rs2127387	5	76532571	PDE8B	a	g	0.144	0.139	0.030	<0.001	<0.001	28	0.146	0.030	<0.001	<0.001	-0.028	0.145	0.85	0.95
rs7702192	5	76554807	PDE8B	a	c	0.070	0.043	0.028	0.13	0.48	0	0.047	0.029	0.11	0.42	-0.043	0.143	0.77	0.95
rs113974964	5	76652403	PDE8B	t	c	-0.124	-0.075	0.077	0.33	0.70	0	-0.081	0.079	0.31	0.66	0.093	0.425	0.83	0.95
rs139149784	5	76660193	PDE8B	a	g	0.156	0.314	0.151	0.04	0.23	0	0.340	0.156	0.03	0.18	-0.097	0.617	0.88	0.95
rs182873197	5	76773148	PDE8B	t	c	-0.080	0.003	0.059	0.97	0.97	0	0.005	0.060	0.93	0.97	-0.127	0.408	0.76	0.95
rs1265091	6	31108129	PSORS1C1	t	c	0.058	-0.026	0.042	0.54	0.81	11	-0.017	0.043	0.69	0.89	-0.243	0.209	0.25	0.95
rs744103	6	43805362	VEGFA/ LOC100132354	a	t	0.092	0.046	0.033	0.16	0.55	0	0.048	0.033	0.15	0.50	-0.012	0.167	0.94	0.95
rs9381266	6	43905037	VEGFA /LOC100132354	t	c	0.073	0.045	0.033	0.17	0.55	0	0.046	0.034	0.18	0.50	0.024	0.180	0.90	0.95
rs9497965	6	148521292	SASH1	t	c	0.044	0.020	0.030	0.51	0.81	0	0.023	0.030	0.45	0.79	-0.054	0.139	0.70	0.95
rs73022105	6	165973757	PDE10A	t	c	0.105	-0.043	0.075	0.56	0.81	0	-0.059	0.077	0.45	0.79	0.314	0.372	0.40	0.95
rs1079418	6	166047034	PDE10A	a	g	0.101	0.100	0.031	<0.01	0.03	22	0.092	0.032	<0.01	0.05	0.259	0.143	0.07	0.83
rs56009477	8	23356964	SLC25A37	a	g	0.050	0.051	0.043	0.24	0.65	0	0.052	0.044	0.23	0.53	0.013	0.217	0.95	0.95
rs2439301	8	32433013	NRG1	a	g	-0.059	-0.019	0.036	0.60	0.83	0	-0.021	0.036	0.56	0.79	0.038	0.181	0.84	0.95

rs10957494	8	70365025	SULF1	a	g	-0.036	-0.028	0.032	0.37	0.72	0	-0.023	0.033	0.49	0.79	-0.160	0.157	0.31	0.95
rs118039499	8	133771635	TG	a	c	0.185	0.139	0.108	0.20	0.59	0	0.145	0.108	0.18	0.50	-0.547	1.195	0.65	0.95
rs2739067	8	133951991	TG	a	g	-0.042	-0.019	0.029	0.53	0.81	0	-0.017	0.030	0.56	0.79	-0.045	0.145	0.76	0.95
rs10814915	9	4290544	GLIS3	t	c	0.042	0.056	0.029	0.05	0.28	5	0.062	0.029	0.04	0.18	-0.081	0.137	0.55	0.95
rs9298749	9	16214340	C9orf92	a	c	-0.038	0.029	0.031	0.35	0.70	0	0.028	0.031	0.37	0.75	0.063	0.178	0.72	0.95
rs8176645	9	136149098	ABO	a	t	0.052	0.092	0.031	<0.01	0.04	0	0.090	0.031	<0.01	0.05	0.155	0.158	0.33	0.95
rs11255790	10	8682180	GATA3	t	c	-0.039	-0.012	0.032	0.71	0.90	32	-0.019	0.033	0.56	0.79	0.203	0.179	0.26	0.95
rs4933466	10	89849519	PTEN	a	g	0.037	0.029	0.029	0.32	0.70	0	0.025	0.030	0.40	0.75	0.141	0.155	0.37	0.95
rs12284404	11	45228686	PRDM11	a	g	-0.067	-0.074	0.032	0.02	0.19	0	-0.071	0.033	0.03	0.18	-0.132	0.142	0.36	0.95
rs4445669	11	115045237	CADM1	t	c	-0.039	-0.032	0.029	0.28	0.68	0	-0.034	0.030	0.26	0.57	0.019	0.136	0.89	0.95
rs7329958	13	24782080	SPATA13	t	c	-0.044	-0.007	0.031	0.82	0.96	0	-0.010	0.032	0.75	0.90	0.076	0.171	0.66	0.95
rs398745	14	36536181	MBIP	a	c	-0.052	-0.031	0.030	0.29	0.68	75	-0.043	0.030	0.15	0.50	0.260	0.148	0.08	0.83
rs2254613	14	36713154	MBIP	t	g	-0.035	-0.004	0.031	0.91	0.97	0	-0.005	0.032	0.88	0.97	0.019	0.143	0.90	0.95
rs11159482	14	81490842	TSHR	t	c	0.085	0.105	0.066	0.11	0.47	85	0.142	0.068	0.04	0.18	-0.667	0.310	0.03	0.67
rs59334515	14	81594143	TSHR	t	c	-0.054	-0.032	0.035	0.35	0.70	75	-0.045	0.035	0.20	0.51	0.338	0.188	0.08	0.83
rs12893151	14	81619945	TSHR	a	c	-0.057	0.016	0.038	0.66	0.88	0	0.021	0.039	0.58	0.79	-0.084	0.174	0.63	0.95
rs8015085	14	93585331	ITPK1	a	g	0.067	0.033	0.039	0.40	0.74	0	0.034	0.040	0.40	0.75	0.019	0.225	0.93	0.95
rs17477923	15	49711185	FAM227B/FGF7	t	c	0.083	-0.010	0.032	0.76	0.93	23	-0.002	0.033	0.95	0.97	-0.184	0.156	0.24	0.95
rs11639111	15	49749735	FAM227B/FGF7	t	c	0.045	0.017	0.029	0.56	0.81	0	0.017	0.030	0.56	0.79	0.018	0.160	0.91	0.95
rs13329353	15	89113877	DET1	t	c	0.061	-0.050	0.032	0.12	0.47	0	-0.050	0.033	0.13	0.49	-0.067	0.153	0.66	0.95
rs1045476	16	4015313	ADCY9	a	g	0.047	0.027	0.041	0.51	0.81	87	0.053	0.042	0.20	0.51	-0.471	0.183	0.01	0.67
rs30227	16	14405428	MIR365A	t	c	-0.046	-0.008	0.031	0.79	0.95	0	-0.012	0.032	0.71	0.89	0.064	0.144	0.66	0.95
rs17767491	16	79745487	MAF	a	g	0.088	-0.011	0.031	0.72	0.90	79	0.003	0.032	0.93	0.97	-0.336	0.153	0.03	0.67
rs77819282	17	44762589	NSF	a	g	0.043	-0.016	0.036	0.65	0.88	61	-0.004	0.037	0.91	0.97	-0.290	0.173	0.10	0.83
rs1157994	17	59338574	BCAS3	a	g	-0.083	0.009	0.077	0.91	0.97	0	0.004	0.080	0.96	0.97	0.075	0.299	0.80	0.95
rs1042673	17	70121339	SOX9	a	g	-0.055	-0.052	0.029	0.08	0.38	0	-0.054	0.030	0.07	0.34	-0.017	0.145	0.91	0.95
rs963384	17	70369758	SOX9	t	c	0.035	-0.017	0.029	0.57	0.81	0	-0.017	0.030	0.58	0.79	-0.018	0.163	0.91	0.95
rs4804413	19	7222655	INSR	t	c	0.053	-0.003	0.029	0.91	0.97	0	-0.005	0.030	0.87	0.97	0.039	0.158	0.81	0.95
rs1203944	20	22596879	FOXA2	t	c	-0.051	-0.041	0.035	0.24	0.65	0	-0.048	0.036	0.18	0.50	0.102	0.166	0.54	0.95
rs12390237	23	3612081	PRKX	a	g	-0.046	-0.103	0.040	0.01	0.10	82	-0.133	0.042	<0.01	0.03	0.184	0.129	0.16	0.95

Supplemental Table 6. FT4 SNPs with FT4 concentrations in cord blood

							Meta-analysis GenR wave 1 and 2					GenR wave 1				GenR wave 2			
SNP	Chr	Position	Locus	A1	A2	b Adults	b	se	pval	p.fdr	I2	b	se	pval	p.fdr	b	se	pval	p.fdr
rs145019385	1	54252139	DIO1	t	c	0.181	0.037	0.136	0.79	0.96	0	0.049	0.138	0.72	0.88	-0.490	0.932	0.60	0.72
rs12033572	1	54369674	DIO1	c	g	0.115	0.095	0.087	0.27	0.63	0	0.083	0.088	0.35	0.67	0.667	0.602	0.27	0.68
rs2235544	1	54375570	DIO1	a	c	0.139	-0.003	0.029	0.93	0.96	26	-0.010	0.029	0.74	0.88	0.155	0.139	0.27	0.68
rs954878	1	54578401	DIO1	a	g	-0.058	0.025	0.029	0.39	0.63	0	0.020	0.030	0.49	0.76	0.151	0.156	0.34	0.68
rs4954192	2	135632981	ACMSD	t	c	-0.033	0.042	0.032	0.18	0.63	0	0.040	0.032	0.21	0.66	0.084	0.151	0.58	0.72
rs6785807	3	181718601	SOX2-OT	a	g	-0.057	-0.036	0.042	0.40	0.63	0	-0.034	0.043	0.43	0.73	-0.059	0.177	0.74	0.85
rs6854291	4	170992760	AADAT	a	g	0.117	-0.135	0.048	0.01	0.15	35	-0.147	0.049	0.00	0.08	0.146	0.231	0.53	0.72
rs10946313	6	19381386	ID4	t	c	0.044	-0.007	0.030	0.83	0.96	0	-0.004	0.031	0.91	0.97	-0.082	0.153	0.59	0.72
rs9356988	6	25777481	SLC17A4	a	g	-0.052	0.002	0.033	0.96	0.96	0	0.001	0.033	0.97	0.97	0.004	0.175	0.98	>0.99
rs137964359	6	26001742	SLC17A4	t	c	-0.200	0.192	0.210	0.36	0.63	8	0.201	0.210	0.34	0.67	-5.185	5.163	0.32	0.68
rs17185536	6	100620931	LOC728012	t	c	0.071	-0.034	0.039	0.38	0.63	0	-0.040	0.039	0.31	0.67	0.147	0.209	0.48	0.72
rs67583169	8	61212179	CA8	c	g	0.062	0.048	0.044	0.27	0.63	72	0.065	0.045	0.14	0.50	-0.354	0.216	0.10	0.68
rs10119187	9	4223660	GLIS3	t	c	0.048	-0.014	0.038	0.72	0.93	46	-0.024	0.039	0.54	0.77	0.253	0.200	0.21	0.68
rs10739496	9	100552559	FOXE1	t	c	0.078	0.053	0.030	0.08	0.41	0	0.050	0.031	0.10	0.45	0.103	0.150	0.49	0.72
rs10984606	9	100739117	FOXE1	t	g	-0.040	0.022	0.028	0.43	0.63	31	0.029	0.029	0.31	0.67	-0.149	0.145	0.31	0.68
rs10818937	9	127015440	NEK6	t	c	-0.039	-0.053	0.031	0.09	0.41	0	-0.055	0.032	0.09	0.45	0.000	0.145	1.00	>0.99
rs4842131	9	139092679	LHX3	t	c	-0.104	-0.077	0.032	0.02	0.28	58	-0.088	0.033	0.01	0.13	0.142	0.145	0.33	0.68
rs55679545	9	139122363	LHX3	a	g	0.044	-0.045	0.043	0.29	0.63	0	-0.038	0.044	0.38	0.70	-0.179	0.194	0.36	0.68
rs11039355	11	47737501	FNBP4	t	c	-0.039	0.005	0.030	0.86	0.96	0	0.010	0.031	0.73	0.88	-0.130	0.156	0.41	0.68
rs4149056	12	21331549	SLCO1B1	t	c	-0.048	0.046	0.038	0.23	0.63	0	0.041	0.040	0.30	0.67	0.132	0.159	0.41	0.68
rs150816132	14	80464293	DIO2	a	g	-0.220	0.222	0.160	0.17	0.63	59	0.280	0.165	0.09	0.45	-0.851	0.707	0.23	0.68
rs978055	14	80534869	DIO2	a	t	0.038	0.024	0.029	0.40	0.63	14	0.018	0.030	0.54	0.77	0.184	0.151	0.23	0.68
rs225014	14	80669580	DIO2	t	c	0.047	-0.028	0.029	0.35	0.63	0	-0.029	0.030	0.34	0.67	0.006	0.156	0.97	>0.99
rs12323871	14	101852075	DIO3OS	t	c	-0.047	-0.025	0.037	0.49	0.69	0	-0.021	0.037	0.58	0.78	-0.175	0.213	0.41	0.68
rs11626434	14	101998443	DIO3OS	c	g	0.053	0.066	0.031	0.03	0.33	59	0.056	0.031	0.07	0.45	0.303	0.156	0.06	0.68
rs12907106	15	63873658	USP3	c	g	-0.039	0.003	0.033	0.93	0.96	0	0.002	0.033	0.96	0.97	0.038	0.173	0.83	0.91
rs8063103	16	12703395	SNX29	c	g	-0.051	0.007	0.041	0.86	0.96	0	0.004	0.041	0.92	0.97	0.140	0.252	0.58	0.72
rs11078333	17	16049626	NCOR1	a	t	0.042	0.053	0.029	0.07	0.41	48	0.046	0.029	0.12	0.45	0.277	0.164	0.09	0.68
rs1080094	18	29173795	SLC25A52	a	g	-0.042	-0.016	0.029	0.59	0.79	28	-0.009	0.030	0.76	0.88	-0.190	0.150	0.21	0.68
rs113107469	18	29306737	SLC25A52	t	c	0.200	-0.072	0.080	0.36	0.63	0	-0.061	0.081	0.45	0.73	-0.379	0.429	0.38	0.68
rs56069042	18	57914644	MC4R	a	g	0.099	-0.136	0.075	0.07	0.41	0	-0.129	0.076	0.09	0.45	-0.536	0.565	0.35	0.68

Supplemental Table 7. Associations of TSH and FT4 PRS with thyroid function in cord blood

		b	se	pval	I2 (meta) or Expl. Var. (cohort)	N
**Meta**	TSH	0.09	0.01	<0.001	90	2388
	FT4	0.00	0.01	0.88	0	2374
**GenR wave 1**	TSH	0.10	0.01	<0.001	0.018	2280
	FT4	0.00	0.01	0.95	0.000	2267
**GenR wave 2**	TSH	-0.06	0.05	0.27	0.012	108
	FT4	0.06	0.06	0.29	0.011	107

Supplemental Table 8. Associations of TSH unweighted PRS with thyroid function in childhood

		b	se	pval	I2 (meta) or Expl. Var. (cohort)	N
**Meta**	TSH	0.15	0.01	<0.001	0	7231
	FT4	-0.03	0.01	<0.001	31	7206
**GenR wave 1**	TSH	0.14	0.02	<0.001	0.049	1542
	FT4	-0.05	0.02	0.01	0.005	1534
**GenR wave 2**	TSH	0.16	0.02	<0.001	0.066	627
	FT4	-0.04	0.02	0.11	0.004	625
**ALSPAC**	TSH	0.14	0.01	<0.001	0.043	3382
	FT4	-0.02	0.01	0.04	0.001	3367
**BLTS**	TSH	0.16	0.02	<0.001	0.059	1680
	FT4	-0.06	0.02	<0.01	0.006	1680

Supplemental Table 9. Associations of FT4 unweighted PRS with thyroid function in childhood

		b	se	pval	I2 (meta) or Expl. Var.	N
**Meta**	FT4	0.09	0.01	<0.001	0	7142
	FT4 normal range	0.08	0.01	<0.001	45	7057
	TSH	0.03	0.01	<0.001	0	7231
	FT3	-0.02	0.01	0.02	0	5023
**GenR wave 1**	FT4	0.10	0.02	<0.001	0.024	1534
	FT4 normal range	0.11	0.02	<0.001	0.029	1470
	TSH	0.02	0.02	0.18	0.001	1542
**GenR wave 2**	FT4	0.11	0.03	<0.001	0.030	625
	FT4 normal range	0.10	0.03	<0.001	0.023	590
	TSH	0.01	0.03	0.58	0.000	627
**ALSPAC**	FT4	0.09	0.01	<0.001	0.015	3367
	FT4 normal range	0.07	0.01	<0.001	0.010	3222
	TSH	0.04	0.01	<0.01	0.003	3382
	FT3	-0.02	0.01	0.07	0.000	3343
**BLTS**	FT4	0.07	0.02	<0.001	0.011	1680
	FT4 normal range	0.08	0.02	<0.001	0.011	1680
	TSH	0.03	0.02	0.122	0.002	1680
	FT3	-0.02	0.02	0.290	0.001	1680

Supplemental Table 10. FT4 SNPs with FT3 concentrations in separate cohorts

						ALSPAC				BLTS			
SNP	Chr	Position	Locus	A1	A2	b	se	pval	p.fdr	b	se	pval	p.fdr
rs145019385	1	54252139	DIO1	t	c	0.095	0.084	0.26	0.44	-0.096	0.126	0.45	0.68
rs12033572	1	54369674	DIO1	c	g	0.088	0.061	0.15	0.36	-0.125	0.089	0.16	0.50
rs2235544	1	54375570	DIO1	a	c	0.183	0.024	<0.001	<0.001	-0.114	0.037	<0.01	0.05
rs954878	1	54578401	DIO1	a	g	-0.080	0.024	<0.01	0.01	0.029	0.038	0.44	0.68
rs4954192	2	1,36E+08	ACMSD	t	c	-0.044	0.025	0.08	0.22	-0.053	0.036	0.14	0.49
rs6785807	3	1,82E+08	SOX2-OT	a	g	-0.040	0.033	0.22	0.44	0.005	0.049	0.93	0.93
rs6854291	4	1,71E+08	AADAT	a	g	0.214	0.043	<0.001	<0.001	-0.135	0.063	0.03	0.16
rs10946313	6	19381386	ID4	t	c	0.018	0.025	0.48	0.65	0.033	0.037	0.37	0.64
rs9356988	6	25777481	SLC17A4	a	g	-0.018	0.028	0.50	0.65	0.026	0.042	0.54	0.72
rs137964359	6	26001742	SLC17A4	t	c	-0.075	0.097	0.44	0.62	0.029	0.176	0.87	0.93
rs17185536	6	1,01E+08	LOC728012	t	c	0.079	0.030	0.01	0.03	0.058	0.044	0.19	0.54
rs67583169	8	61212179	CA8	c	g	0.013	0.036	0.72	0.79	0.013	0.053	0.81	0.92
rs10119187	9	4223660	GLIS3	t	c	-0.021	0.033	0.53	0.65	-0.027	0.049	0.58	0.72
rs10739496	9	1,01E+08	FOXE1	t	c	0.054	0.026	0.04	0.12	-0.108	0.040	0.01	0.06
rs10984606	9	1,01E+08	FOXE1	t	g	0.020	0.024	0.40	0.62	0.109	0.037	<0.01	0.05
rs10818937	9	1,27E+08	NEK6	t	c	0.005	0.026	0.84	0.90	0.036	0.040	0.37	0.64
rs4842131	9	1,39E+08	LHX3	t	c	-0.071	0.026	0.01	0.03	-0.027	0.037	0.46	0.68
rs55679545	9	1,39E+08	LHX3	a	g	0.015	0.029	0.62	0.74	0.026	0.040	0.52	0.72
rs11039355	11	47737501	FNBP4	t	c	-0.039	0.026	0.14	0.36	0.063	0.039	0.11	0.41
rs4149056	12	21331549	SLCO1B1	t	c	-0.041	0.035	0.24	0.44	-0.028	0.051	0.58	0.72
rs150816132	14	80464293	DIO2	a	g	-0.384	0.134	<0.01	0.02	0.201	0.161	0.21	0.55
rs978055	14	80534869	DIO2	a	t	-0.033	0.025	0.19	0.41	0.044	0.038	0.25	0.59
rs225014	14	80669580	DIO2	t	c	0.001	0.025	0.98	0.98	-0.012	0.039	0.77	0.91
rs12323871	14	1,02E+08	DIO3OS	t	c	-0.066	0.031	0.04	0.12	-0.046	0.049	0.35	0.64
rs11626434	14	1,02E+08	DIO3OS	c	g	0.073	0.026	0.01	0.02	-0.038	0.038	0.33	0.64
rs12907106	15	63873658	USP3	c	g	-0.021	0.027	0.43	0.62	0.040	0.041	0.32	0.64
rs8063103	16	12703395	SNX29	c	g	0.002	0.035	0.95	0.98	-0.148	0.054	0.01	0.06
rs11078333	17	16049626	NCOR1	a	t	0.027	0.025	0.27	0.44	0.090	0.038	0.02	0.10
rs1080094	18	29173795	SLC25A52	a	g	0.011	0.025	0.66	0.76	-0.008	0.037	0.83	0.92
rs113107469	18	29306737	SLC25A52	t	c	0.339	0.066	<0.001	<0.001	-0.010	0.100	0.92	0.93
rs56069042	18	57914644	MC4R	a	g	0.079	0.070	0.26	0.44	0.177	0.107	0.10	0.41

Supplemental Table 11. Meta-analysis FT4 SNPs with FT4 concentrations in the normal range of FT4

SNP	Chr	Position	Locus	A1	A2	b Adults	b	Deviation from adults in %	se	pval	p.fdr	I2	CQp
rs145019385	1	54252139	DIO1	t	c	0.181	0.067	-63.0	0.063	0.29	0.37	0	0.53
rs12033572	1	54369674	DIO1	c	g	0.115	0.16	39.1	0.046	<0.001	<0.01	22	0.28
rs2235544	1	54375570	DIO1	a	c	0.139	0.17	22.3	0.017	<0.001	<0.001	52	0.10
rs954878	1	54578401	DIO1	a	g	-0.058	-0.045	-22.4	0.017	0.01	0.03	60	0.06
rs4954192	2	1,36E+08	ACMSD	t	c	-0.033	-0.036	-12.2	0.018	<0.05	0.11	0	0.41
rs6785807	3	1,82E+08	SOX2-OT	a	g	-0.057	-0.03	-49.2	0.023	0.19	0.30	0	0.68
rs6854291	4	1,71E+08	AADAT	a	g	0.117	0.201	71.8	0.030	<0.001	<0.001	66	0.03
rs10946313	6	19381386	ID4	t	c	0.044	0.016	-65.2	0.018	0.37	0.44	28	0.25
rs9356988	6	25777481	SLC17A4	a	g	-0.052	-0.054	5.9	0.019	0.01	0.02	0	0.76
rs137964359	6	26001742	SLC17A4	t	c	-0.200	-0.116	-42.0	0.083	0.16	0.30	0	0.99
rs17185536	6	1,01E+08	LOC728012	t	c	0.071	0.042	-42.5	0.021	0.05	0.11	32	0.22
rs67583169	8	61212179	CA8	c	g	0.062	0.008	-86.9	0.025	0.76	0.76	0	0.54
rs10119187	9	4223660	GLIS3	t	c	0.048	-0.026	-152.0	0.023	0.26	0.37	23	0.27
rs10739496	9	1,01E+08	FOXE1	t	c	0.078	0.03	-61.5	0.018	0.11	0.21	0	0.96
rs10984606	9	1,01E+08	FOXE1	t	g	-0.040	0.022	-155.0	0.017	0.20	0.30	0	0.94
rs10818937	9	1,27E+08	NEK6	t	c	-0.039	-0.016	-66.7	0.019	0.39	0.45	0	0.49
rs4842131	9	1,39E+08	LHX3	t	c	-0.104	-0.084	-19.2	0.018	<0.001	<0.001	0	0.63
rs55679545	9	1,39E+08	LHX3	a	g	0.044	0.021	-52.3	0.021	0.31	0.38	0	0.68
rs11039355	11	47737501	FNBP4	t	c	-0.039	-0.036	-7.7	0.018	0.05	0.11	0	0.46
rs4149056	12	21331549	SLCO1B1	t	c	-0.048	-0.031	-39.2	0.024	0.20	0.30	80	0.00
rs150816132	14	80464293	DIO2	a	g	-0.220	-0.292	32.7	0.090	<0.01	0.01	0	0.69
rs978055	14	80534869	DIO2	a	t	0.038	0.01	-73.7	0.018	0.56	0.60	72	0.01
rs225014	14	80669580	DIO2	t	c	0.047	0.01	-81.5	0.018	0.56	0.60	0	0.56
rs12323871	14	1,02E+08	DIO3OS	t	c	-0.047	-0.046	-2.1	0.023	0.04	0.11	0	0.42
rs11626434	14	1,02E+08	DIO3OS	c	g	0.053	0.084	44.8	0.018	<0.001	<0.001	26	0.26
rs12907106	15	63873658	USP3	c	g	-0.039	-0.021	-48.8	0.019	0.27	0.37	0	0.89
rs8063103	16	12703395	SNX29	c	g	-0.051	-0.033	-36.5	0.025	0.18	0.30	32	0.22
rs11078333	17	16049626	NCOR1	a	t	0.042	0.046	-9.8	0.017	0.01	0.03	15	0.32
rs1080094	18	29173795	SLC25A52	a	g	-0.042	0.007	-116.7	0.018	0.71	0.74	0	0.68
rs113107469	18	29306737	SLC25A52	t	c	0.200	0.107	-46.5	0.047	0.02	0.07	11	0.34
rs56069042	18	57914644	MC4R	a	g	0.099	0.12	13.2	0.047	0.01	0.04	0	0.50

Supplemental Table 12. FT4 SNPs with FT4 concentrations in the normal range of FT4 in separate cohorts

							GenR wave 1				GenR wave 2				ALSPAC				BLTS			
SNP	Chr	Position	Locus	A1	A2	b Adults	b	se	pval	p.fdr	b	se	pval	p.fdr	b	se	pval	p.fdr	b	se	pval	p.fdr
rs145019385	1	54252139	DIO1	t	c	0.181	0.229	0.172	0.18	0.47	-0.150	0.237	0.53	0.89	0.093	0.086	0.28	0.41	-0.014	0.124	0.91	0.93
rs12033572	1	54369674	DIO1	c	g	0.115	0.352	0.114	<0.01	0.02	0.278	0.244	0.26	0.58	0.110	0.063	0.08	0.18	0.128	0.088	0.15	0.46
rs2235544	1	54375570	DIO1	a	c	0.139	0.232	0.036	<0.001	<0.001	0.125	0.060	0.04	0.23	0.177	0.025	<0.001	<0.001	0.110	0.037	<0.01	0.09
rs954878	1	54578401	DIO1	a	g	-0.058	-0.010	0.037	0.79	0.87	-0.081	0.062	0.19	0.57	-0.086	0.025	<0.001	<0.01	0.025	0.037	0.50	0.80
rs4954192	2	1.36E+08	ACMSD	t	c	-0.033	-0.066	0.042	0.11	0.32	0.032	0.062	0.61	0.89	-0.052	0.026	0.04	0.17	-0.004	0.036	0.90	0.93
rs6785807	3	1.82E+08	SOX2-OT	a	g	-0.057	-0.059	0.054	0.28	0.50	-0.103	0.077	0.18	0.57	-0.013	0.034	0.69	0.79	-0.015	0.048	0.76	0.91
rs6854291	4	1.71E+08	AADAT	a	g	0.117	0.301	0.065	<0.001	<0.001	0.396	0.101	<0.001	<0.01	0.157	0.044	<0.001	<0.01	0.119	0.062	0.06	0.34
rs10946313	6	19381386	ID4	t	c	0.044	0.060	0.038	0.11	0.32	0.093	0.061	0.13	0.57	-0.006	0.026	0.81	0.87	-0.009	0.037	0.80	0.92
rs9356988	6	25777481	SLC17A4	a	g	-0.052	-0.081	0.041	<0.05	0.17	-0.088	0.068	0.20	0.57	-0.050	0.028	0.08	0.18	-0.025	0.041	0.54	0.80
rs137964359	6	26001742	SLC17A4	t	c	-0.200	-0.101	0.268	0.71	0.84	0.268	1.034	0.80	0.90	-0.123	0.100	0.22	0.34	-0.111	0.182	0.54	0.80
rs17185536	6	1.01E+08	LOC728012	t	c	0.071	0.020	0.050	0.68	0.84	0.018	0.082	0.83	0.90	0.085	0.030	0.01	0.03	-0.022	0.043	0.61	0.86
rs67583169	8	61212179	CA8	c	g	0.062	0.068	0.056	0.23	0.50	-0.056	0.087	0.52	0.89	-0.014	0.036	0.69	0.79	0.025	0.053	0.64	0.86
rs10119187	9	4223660	GLIS3	t	c	0.048	-0.034	0.048	0.48	0.71	0.102	0.082	0.21	0.57	-0.059	0.034	0.08	0.18	0.007	0.048	0.89	0.93
rs10739496	9	1.01E+08	FOXE1	t	c	0.078	0.035	0.039	0.37	0.61	0.010	0.064	0.87	0.90	0.036	0.026	0.17	0.31	0.016	0.040	0.68	0.88
rs10984606	9	1.01E+08	FOXE1	t	g	-0.040	0.007	0.037	0.85	0.88	0.036	0.059	0.55	0.89	0.031	0.025	0.22	0.34	0.013	0.037	0.73	0.90
rs10818937	9	1.27E+08	NEK6	t	c	-0.039	-0.039	0.040	0.33	0.56	0.030	0.068	0.66	0.89	0.005	0.026	0.86	0.89	-0.056	0.040	0.16	0.46
rs4842131	9	1.39E+08	LHX3	t	c	-0.104	-0.113	0.041	0.01	0.04	-0.031	0.061	0.61	0.89	-0.093	0.027	<0.01	<0.01	-0.064	0.036	0.08	0.34
rs55679545	9	1.39E+08	LHX3	a	g	0.044	-0.024	0.054	0.66	0.84	0.025	0.070	0.72	0.90	0.045	0.030	0.14	0.26	0.003	0.040	0.93	0.93
rs11039355	11	47737501	FNBP4	t	c	-0.039	0.007	0.039	0.86	0.88	-0.039	0.066	0.56	0.89	-0.033	0.027	0.22	0.34	-0.080	0.038	0.04	0.28
rs4149056	12	21331549	SLCO1B1	t	c	-0.048	0.024	0.050	0.63	0.84	-0.256	0.077	<0.01	0.01	-0.060	0.035	0.09	0.19	0.072	0.051	0.15	0.46
rs150816132	14	80464293	DIO2	a	g	-0.220	-0.413	0.194	0.03	0.15	-0.444	0.326	0.17	0.57	-0.316	0.144	0.03	0.12	-0.148	0.157	0.35	0.76
rs978055	14	80534869	DIO2	a	t	0.038	0.050	0.039	0.20	0.47	0.062	0.062	0.32	0.65	-0.048	0.025	0.06	0.17	0.082	0.037	0.03	0.28
rs225014	14	80669580	DIO2	t	c	0.047	0.043	0.039	0.27	0.50	0.014	0.062	0.82	0.90	-0.015	0.026	0.56	0.72	0.034	0.038	0.37	0.76
rs12323871	14	1.02E+08	DIO3OS	t	c	-0.047	-0.053	0.047	0.26	0.50	0.106	0.094	0.26	0.58	-0.060	0.032	0.06	0.17	-0.045	0.049	0.36	0.76
rs11626434	14	1.02E+08	DIO3OS	c	g	0.053	0.113	0.040	<0.01	0.04	0.173	0.066	0.01	0.09	0.051	0.026	0.05	0.17	0.097	0.038	0.01	0.15
rs12907106	15	63873658	USP3	c	g	-0.039	-0.020	0.042	0.63	0.84	-0.025	0.068	0.72	0.90	-0.008	0.028	0.77	0.85	-0.047	0.040	0.24	0.62
rs8063103	16	12703395	SNX29	c	g	-0.051	-0.039	0.054	0.47	0.71	-0.199	0.089	0.03	0.20	0.001	0.036	0.98	0.98	-0.045	0.054	0.40	0.77
rs11078333	17	16049626	NCOR1	a	t	0.042	0.093	0.037	0.01	0.06	-0.007	0.065	0.91	0.91	0.022	0.025	0.38	0.51	0.067	0.037	0.07	0.34
rs1080094	18	29173795	SLC25A52	a	g	-0.042	-0.002	0.038	0.97	0.97	0.009	0.060	0.88	0.90	0.026	0.026	0.31	0.44	-0.028	0.037	0.45	0.79
rs113107469	18	29306737	SLC25A52	t	c	0.200	0.028	0.102	0.78	0.87	-0.079	0.172	0.65	0.89	0.191	0.069	0.01	0.03	0.073	0.097	0.46	0.79
rs56069042	18	57914644	MC4R	a	g	0.099	0.187	0.092	0.04	0.17	0.193	0.158	0.22	0.57	0.037	0.072	0.61	0.76	0.177	0.104	0.09	0.34

Supplemental Table 13. Associations of different TSH PRS with TSH concentrations and different FT4 PRS with FT4 concentrations

			ALSPAC TSH N=3382, FT4 N=3367				Generation R wave 1 TSH N=1542, FT4 N=1534				BLTS N=1680			
		# SNPs	b	se	pval	Expl. Var.	b	se	pval	Expl. Var.	b	se	pval	Expl. Var.
**Normal PRS**	TSH	60	0.15	0.01	<0.001	0.053	0.18	0.02	<0.001	0.057	0.17	0.02	<0.001	0.067
	FT4	31	0.13	0.01	<0.001	0.029	0.14	0.02	<0.001	0.042	0.09	0.02	<0.001	0.015
**β's ALSPAC**	TSH	60	-	-	-	-	0.17	0.02	<0.001	0.067	0.16	0.02	<0.001	0.050
	FT4	31	-	-	-	-	0.17	0.02	<0.001	0.043	0.08	0.02	<0.001	0.012
**β's GenR wave 1**	TSH	60	0.17	0.01	<0.001	0.051	-	-	-	-	0.15	0.02	<0.001	0.046
	FT4	31	0.12	0.01	<0.001	0.025	-	-	-	-	0.08	0.02	<0.001	0.018
**β's BLTS**	TSH	60	0.15	0.01	<0.001	0.037	0.16	0.02	<0.001	0.048	-	-	-	-
	FT4	31	0.10	0.01	<0.001	0.015	0.14	0.02	<0.001	0.041	-	-	-	-
**β's within +/-30% deviation from adults in meta-analysis**	TSH	29	0.12	0.01	<0.001	0.034	0.13	0.02	<0.001	0.040	0.11	0.02	<0.001	0.034
	FT4	9	0.13	0.01	<0.001	0.024	0.12	0.02	<0.001	0.032	0.06	0.02	<0.001	0.010
**SNPs *p*.fdr <0.05 in meta-analysis**	TSH	30	0.16	0.01	<0.001	0.065	0.16	0.01	<0.001	0.074	0.18	0.02	<0.001	0.070
	FT4	11	0.15	0.01	<0.001	0.037	0.17	0.02	<0.001	0.054	0.09	0.02	<0.001	0.020
**SNPs I2<30**	TSH	43	0.12	0.01	<0.001	0.038	0.13	0.02	<0.001	0.036	0.15	0.02	<0.001	0.057
	FT4	22	0.07	0.01	<0.001	0.008	0.09	0.02	<0.001	0.015	0.07	0.02	<0.001	0.010
**SNPs *p* heterogeneity >0.05**	TSH	57	0.14	0.01	<0.001	0.045	0.16	0.02	<0.001	0.045	0.16	0.02	<0.001	0.068
	FT4	26	0.07	0.01	<0.001	0.008	0.10	0.02	<0.001	0.017	0.07	0.02	<0.001	0.011

Abbreviations: I2= percentage of variance that is attributable to study heterogeneity

Supplemental Table 14. Overview new TSH SNPs

SNP	Chr	Position	Locus	A1	A2	AF1 GenR wave 1	AF1 GenR wave 2	AF1 BLTS
rs12743883	1	22513011	*WNT4;MIR4418*	G	A	0.63	0.61	0.63
rs11583886	1	51451499	*CDKN2C;MIR4421*	A	G	0.29	0.28	0.31
rs12029562	1	68166425	*GNG12*	A	G	0.52	0.51	0.55
rs2993047	1	218685055	*C1orf143;MIR548F3*	A	G	0.58	0.58	0.60
rs6721104	2	25994220	*ASXL2*	C	A	0.03	0.03	0.03
rs10186921	2	43644556	*THADA*	T	C	0.54	0.56	0.56
rs62174422	2	169554118	*CERS6*	G	T	0.04	0.04	0.04
rs6717283	2	242516105	*BOK;THAP4*	G	A	0.12	0.13	0.14
rs9865818	3	188072513	*LPP*	G	A	0.42	0.42	0.40
rs4571283	4	177705862	*VEGFC*	A	G	0.86	0.91	0.89
rs77994712	5	58373418	*PDE4D*	G	C	0.05	0.05	0.06
rs751171	6	168819800	*DACT2;SMOC2*	C	T	0.35	0.33	0.33
rs4719486	7	2329497	*SNX8*	A	G	0.43	0.44	0.44
rs700750	7	46753491	*LOC730338;TNS3*	A	C	0.61	0.62	0.64
rs2979181	8	8323088	*PRAG1;CLDN23*	T	A	0.49	0.49	0.49
rs72682433	8	120112818	*COLEC10*	C	T	0.09	0.08	0.10
rs1045774	9	127032607	*NEK6*	G	A	0.38	0.36	0.41
rs546738875	12	549670	*CCDC77*	G	C	-	-	0.001
rs7966590	12	570840	*B4GALNT3*	A	G	0.55	0.57	0.57
rs145153320	12	665822	*B4GALNT3*	T	C	-	-	-
rs10878986	12	69831694	*YEATS4;FRS2*	C	T	0.40	0.38	0.37
rs3184504	12	111884608	*SH2B3*	C	T	0.53	0.53	0.51
rs4393429	13	111191813	*RAB20*	C	T	0.26	0.26	0.27
rs74888443	15	36001394	*DPH6-AS1*	T	C	0.05	0.04	0.05
rs35587648	17	47418178	*ZNF652*	A	G	0.36	0.35	0.36
rs1801690	17	64208285	*APOH*	G	C	0.06	0.06	0.05

rs72978712	19	2634823	*GNG7*	C	T	0.21	0.22	0.20
rs10421676	19	55772412	*PPP6R1;HSPBP1*	G	A	0.62	0.58	0.61
rs6085658	20	6685377	*CASC20;LINC01713*	T	C	0.42	0.38	0.39
rs5997969	22	31790014	*LINC01521;DRG1*	C	T	0.66	0.62	0.65

rs11131761 is a proxy for rs4541283) in BLTS

rs546738875 is not available in Generation R

rs145153320 is not available in Generation R and wildtype for all individuals in BLTS

Abbreviations: SNP= Single Nucleotide Polymorphism, Chr= Chromosome, A1= Allele 1, A2= Allele 2, AF1= Allele frequency of allele 1

Supplemental Table 15. 28 new TSH SNPs with TSH concentrations in childhood in Generation R and BLTS

								Meta-analysis							GenR wave 1				GenR wave 2				BLTS			
SNP	Chr	Position	Nearest gene(s)	A1	A2	b adults	se adults	est	Deviation from adults in %	se	p	p.fdr	I2	CQp	est	se	p	p.fdr	est	se	p	p.fdr	est	se	p	p.fdr
rs12743883	1	22513011	*WNT4;MIR4418*	G	A	0.031	0.005	0.017	-45.2	0.024	0.49	0.86	0	0.44	0.013	0.038	0.73	0.98	-0.045	0.060	0.45	0.88	0.045	0.037	0.23	0.50
rs11583886	1	51451499	*CDKN2C;MIR4421*	A	G	-0.035	0.005	0.007	-120.0	0.026	0.80	0.86	16	0.30	0.012	0.040	0.76	0.98	-0.086	0.067	0.20	0.88	0.033	0.039	0.40	0.59
rs12029562	1	68166425	*GNG12*	A	G	-0.030	0.005	-0.010	-66.7	0.023	0.68	0.86	63	0.07	-0.005	0.036	0.89	0.98	0.104	0.059	0.08	0.75	-0.058	0.036	0.11	0.35
rs2993047	1	218685055	*C1orf143;MIR548F3*	A	G	-0.033	0.005	-0.036	9.1	0.024	0.14	0.39	35	0.21	-0.001	0.038	0.98	0.98	0.006	0.060	0.92	0.98	-0.085	0.037	0.02	0.24
rs6721104	2	25994220	*ASXL2*	C	A	0.087	0.012	0.001	-98.9	0.071	0.99	0.99	67	<0.05	0.203	0.112	0.07	0.45	-0.259	0.179	0.15	0.88	-0.094	0.108	0.39	0.59
rs10186921	2	43644556	*THADA*	T	C	0.040	0.005	0.016	-60.0	0.024	0.50	0.86	23	0.28	-0.002	0.037	0.97	0.98	0.101	0.058	0.08	0.75	-0.001	0.037	0.99	0.99
rs62174422	2	169554118	*CERS6*	G	T	-0.083	0.012	-0.127	53.0	0.062	0.04	0.19	0	0.38	-0.158	0.097	0.10	0.47	0.068	0.154	0.66	0.88	-0.170	0.095	0.07	0.29
rs6717283	2	242516105	*BOK;THAP4*	G	A	0.046	0.007	-0.011	-123.9	0.034	0.74	0.86	0	0.67	-0.015	0.053	0.78	0.98	0.056	0.085	0.51	0.88	-0.034	0.053	0.52	0.72
rs9865818	3	188072513	*LPP*	G	A	-0.026	0.005	0.004	-115.4	0.024	0.88	0.92	0	0.87	0.006	0.037	0.88	0.98	-0.025	0.060	0.67	0.88	0.012	0.037	0.75	0.87
rs4571283	4	177705862	*VEGFC*	A	G	0.045	0.008	0.088	95.6	0.040	0.03	0.17	23	0.27	0.134	0.061	0.03	0.28	-0.105	0.136	0.44	0.88	0.082	0.058	0.16	0.45
rs77994712	5	58373418	*PDE4D*	G	C	-0.066	0.011	-0.056	-15.2	0.051	0.27	0.62	0	0.90	-0.029	0.081	0.72	0.98	-0.056	0.140	0.69	0.88	-0.079	0.073	0.28	0.56
rs751171	6	168819800	*DACT2;SMOC2*	C	T	0.033	0.005	-0.006	-118.2	0.025	0.80	0.86	0	0.51	0.008	0.038	0.84	0.98	0.041	0.064	0.52	0.88	-0.037	0.038	0.33	0.56
rs4719486	7	2329497	*SNX8*	A	G	-0.027	0.005	-0.069	155.6	0.024	<0.01	0.04	28	0.25	-0.098	0.036	0.01	0.14	0.023	0.063	0.72	0.88	-0.069	0.037	0.06	0.29
rs700750	7	46753491	*LOC730338;TNS3*	A	C	0.034	0.005	0.012	-64.7	0.024	0.62	0.86	8	0.34	-0.002	0.037	0.96	0.98	-0.046	0.058	0.43	0.88	0.050	0.037	0.18	0.46
rs2979181	8	8323088	*PRAG1;CLDN23*	T	A	-0.036	0.005	-0.036	0.0	0.024	0.14	0.39	0	0.85	-0.020	0.036	0.57	0.98	-0.048	0.059	0.42	0.88	-0.047	0.037	0.21	0.48
rs72682433	8	120112818	*COLEC10*	C	T	0.042	0.008	0.057	35.7	0.040	0.15	0.39	0	0.50	0.002	0.062	0.97	0.98	0.069	0.108	0.53	0.88	0.102	0.059	0.08	0.29
rs1045774	9	127032607	*NEK6*	G	A	0.032	0.005	0.007	-78.1	0.024	0.77	0.86	0	0.68	0.032	0.038	0.39	0.98	-0.022	0.059	0.71	0.88	-0.005	0.038	0.88	0.92
rs546738875	12	549670	*CCDC77*	G	C	0.390	0.061	NA	NA	NA	NA	NA	NA	NA	NA	NA	NA	NA	NA	NA	NA	NA	-1.348	0.607	0.03	0.19
rs7966590	12	570840	*B4GALNT3*	A	G	0.039	0.005	0.088	125.6	0.027	<0.001	0.04	0	0.46	0.141	0.051	0.01	0.14	0.075	0.072	0.30	0.88	0.064	0.036	0.07	0.29
rs10878986	12	69831694	*YEATS4;FRS2*	C	T	-0.027	0.005	-0.047	74.1	0.024	<0.05	0.19	35	0.22	-0.009	0.037	0.81	0.98	-0.022	0.057	0.70	0.88	-0.097	0.037	<0.01	0.24
rs3184504	12	111884608	*SH2B3*	C	T	-0.030	0.005	0.013	-143.3	0.024	0.59	0.86	1	0.36	0.050	0.036	0.16	0.50	0.004	0.058	0.94	0.98	-0.023	0.037	0.54	0.72
rs4393429	13	111191813	*RAB20*	C	T	-0.030	0.005	-0.010	-66.7	0.026	0.69	0.86	0	0.89	0.004	0.040	0.92	0.98	-0.015	0.065	0.82	0.96	-0.023	0.041	0.57	0.72
rs74888443	15	36001394	*DPH6-AS1*	T	C	0.060	0.011	0.168	180.0	0.057	<0.01	0.04	24	0.27	0.082	0.091	0.36	0.98	0.378	0.160	0.02	0.56	0.182	0.082	0.03	0.24

rs35587648	17	47418178	*ZNF652*	A	G	0.032	0.005	0.053	65.6	0.025	0.03	0.17	0	0.59	0.055	0.038	0.15	0.50	-0.002	0.062	0.98	0.98	0.072	0.038	0.06	0.29
rs1801690	17	64208285	*APOH*	G	C	-0.065	0.010	0.019	-129.2	0.052	0.71	0.86	0	0.76	0.014	0.080	0.87	0.98	0.095	0.120	0.43	0.88	-0.012	0.083	0.89	0.92
rs72978712	19	2634823	*GNG7*	C	T	0.044	0.007	-0.017	-138.6	0.032	0.60	0.86	0	0.96	-0.018	0.055	0.74	0.98	-0.039	0.088	0.66	0.88	-0.011	0.045	0.81	0.91
rs10421676	19	55772412	*PPP6R1; HSPBP1*	G	A	0.027	0.005	0.040	48.1	0.024	0.10	0.34	0	0.57	0.065	0.038	0.08	0.45	-0.009	0.059	0.88	0.98	0.036	0.037	0.33	0.56
rs6085658	20	6685377	*CASC20; LINC01713*	T	C	-0.029	0.005	0.017	-158.6	0.024	0.47	0.86	47	0.15	0.056	0.037	0.13	0.50	-0.078	0.058	0.18	0.88	0.017	0.037	0.65	0.79
rs5997969	22	31790014	*LINC01521;DRG1*	C	T	-0.027	0.005	0.021	-177.8	0.025	0.39	0.84	0	0.63	0.026	0.039	0.49	0.98	-0.035	0.064	0.58	0.88	0.035	0.037	0.34	0.56

rs11131761 is a proxy for rs4541283 in BLTS

rs546738875 is not available in Generation R

Supplemental Table 16. Meta-regression of age with effect size and explained variance of FT4 PRS with FT4 concentrations

	Effect size			Explained variance		
	b	se	pval	b	se	pval
Mean age per cohort	-0.01	0.00	0.053	-0.39	0.09	0.046

1. Zhou W, Brumpton B, Kabil O, Gudmundsson J, Thorleifsson G, Weinstock J, Zawistowski M, Nielsen JB, Chaker L, Medici M, Teumer A, Naitza S, Sanna S, Schultheiss UT, Cappola A, Karjalainen J, Kurki M, Oneka M, Taylor P, Fritsche LG, Graham SE, Wolford BN, Overton W, Rasheed H, Haug EB, Gabrielsen ME, Skogholt AH, Surakka I, Davey Smith G, Pandit A, Roychowdhury T, Hornsby WE, Jonasson JG, Senter L, Liyanarachchi S, Ringel MD, Xu L, Kiemeney LA, He H, Netea-Maier RT, Mayordomo JI, Plantinga TS, Hrafnkelsson J, Hjartarson H, Sturgis EM, Palotie A, Daly M, Citterio CE, Arvan P, Brummett CM, Boehnke M, de la Chapelle A, Stefansson K, Hveem K, Willer CJ, Åsvold BO. GWAS of thyroid stimulating hormone highlights pleiotropic effects and inverse association with thyroid cancer. Nat Commun. 2020;11(1):3981.
2. Medina-Gomez C, Felix JF, Estrada K, Peters MJ, Herrera L, Kruithof CJ, Duijts L, Hofman A, van Duijn CM, Uitterlinden AG. Challenges in conducting genome-wide association studies in highly admixed multi-ethnic populations: the Generation R Study. European journal of epidemiology. 2015;30(4):317-30.
3. Taylor AE, Jones HJ, Sallis H, Euesden J, Stergiakouli E, Davies NM, Zammit S, Lawlor DA, Munafò MR, Davey Smith G. Exploring the association of genetic factors with participation in the Avon Longitudinal Study of Parents and Children. International journal of epidemiology. 2018;47(4):1207-16.
4. Medland SE, Nyholt DR, Painter JN, McEvoy BP, McRae AF, Zhu G, Gordon SD, Ferreira MAR, Wright MJ, Henders AK. Common variants in the trichohyalin gene are associated with straight hair in Europeans. The American Journal of Human Genetics. 2009;85(5):750-5.
